# The Founders’ 400 and Chicago Perinatal Origins of Disease study protocol: Following a prospective, longitudinal cohort from early pregnancy through two years of postnatal life

**DOI:** 10.1371/journal.pone.0332928

**Published:** 2025-09-29

**Authors:** Stephanie A. Fisher, Tonia N. Branche, Mary J. Akel, Jessica Dwyer, Gabriella Smith, Aaron Hamvas, Lynn M. Yee, Patrick C. Seed, Leena B. Mithal

**Affiliations:** 1 Department of Obstetrics and Gynecology, Division of Maternal-Fetal Medicine, Feinberg School of Medicine, Northwestern University Feinberg School of Medicine, Chicago, Illinois, United States of America; 2 Department of Pediatrics, Division of Neonatology, Ann and Robert H. Lurie Children’s Hospital, Chicago, Illinois, United States of America; 3 Department of Pediatrics, Division of Infectious Diseases, Ann and Robert H. Lurie Children’s Hospital, Chicago, Illinois, United States of America; 4 Department of Microbiology-Immunology, Northwestern University Feinberg School of Medicine, Chicago, Illinois United States of America; National Research Centre, EGYPT

## Abstract

**Introduction:**

The primary aim of the Chicago Perinatal Origins of Disease (CPOD) study is to characterize social, environmental, and biological exposures from early pregnancy through two years of postnatal life among a diverse cohort of mother-fetus/child dyads in the Chicago metropolitan community and to examine associations with pregnancy and early childhood health outcomes. This study is committed to ensuring the inclusion of participants historically underrepresented in perinatal research and most impacted by perinatal health inequities. CPOD is designed to align with key stakeholder and community input.

**Methods:**

Approximately 400 pregnant people 8–28 weeks gestation and their neonates will be recruited into a longitudinal, prospective observational study enriched for participants who self-identify as Black and/or Latinx. Pregnant participants are followed at three time points antenatally and during their delivery hospitalization; mother-child dyads are followed at five time points in the first two years of life. Semi-structured interviews, patient-reported quantitative surveys, electronic health record abstraction, biological specimens, and environmental sampling from participant homes comprise data collection methods. Biospecimens (including placental biopsies) from mothers, infants, and other household members are collected, processed, and stored in a biorepository. Translational approaches, including a variety of biospecimen analyses (e.g., epigenetics, metabolomics, placental histopathology, microbiome analyses), will be employed to evaluate psychosocial and environmental exposures associated with biologic changes, and how dysregulation of one’s underlying biology during pregnancy and early childhood are associated with adverse health outcomes.

**Discussion:**

CPOD is a unique, prospective, observational study that includes a large, ethnically diverse cohort; rich, multifactorial phenotypic characterization of maternal health and pregnancy outcomes, neonatal health, and early childhood neurobehavior; and development of a biorepository of social, environmental, and clinical data and biospecimens from early pregnancy to two years of postnatal life. Using translational science approaches, data from this cohort will provide clinical and mechanistic insights into how environmental and psychosocial exposures, both during pregnancy and transgenerationally, influence changes in the underlying biology of maternal-child dyads, and how these changes are associated with the risk of adverse health outcomes that contribute to future disease.

## Introduction

Maternal exposures and the *in utero* environment significantly influence pregnancy outcomes, fetal growth, and postnatal development, affecting health throughout the life span [1,2]. These exposures impact physiological adaptions via multiple pathways that include metabolic, neuroendocrine, autonomic, and immune mechanisms and can lead to epigenetic changes and transgenerational effects [3]. The preconception, pregnancy, and infancy periods are critical stages for studying and mitigating lifelong adverse health outcomes [4].

Existing longitudinal cohort studies have investigated environmental and biological exposures in relation to adverse pregnancy outcomes and early childhood neurodevelopment [5,6]. However, these cohorts have enrolled predominantly White individuals of higher socioeconomic status (SES) despite adverse maternal and infant health outcomes disproportionately affecting Black and Latinx individuals. Underrepresentation in research perpetuates health disparities [7].

Racial and ethnic health disparities reflect broader social inequities driven by longstanding exposure to racism and discrimination, not inherent biological differences [7,8]. Structural racism and other social determinants lead to chronic stress and increased allostatic load among Black and Latinx individuals, translating to differential physiologic adaptions and health outcomes [9,10]. The Society for Maternal-Fetal Medicine has called for mechanistic research that ties structural racism to biologic pathophysiology of adverse outcomes, emphasizing the need for multidisciplinary teams that center structurally marginalized voices across translational research [7,11].

The Founders’ 400 and Chicago Perinatal Origins of Disease (CPOD) research initiative responds to this call by developing a large cohort of mother-child dyads, enriched for Black, Latinx, and other participants of color from the Chicagoland community. CPOD aims to collect comprehensive, longitudinal data on social and environmental exposures, obstetric and child health outcomes, and biological samples from early pregnancy through two years of postnatal life. The multidisciplinary team includes specialists from various fields of medicine, epidemiology, data science, and environmental microbiology.

Chicago’s diverse yet, in many areas, still segregated population makes it an ideal setting for this study. Twenty-nine percent of the Chicagoland community identifies as Black, 29% identifies as Hispanic or Latinx, and 7% identifies with two or more races [12]. To ensure diverse inclusion and alignment with community input, the CPOD team engaged pregnant and recently postpartum individuals from various racial and socioeconomic backgrounds *a priori* to understand parental perceptions of perinatal research and home environmental monitoring [13]. This pilot study informed the CPOD study design, addressing themes such as minimizing participant burden, building trust through transparency and engagement, framing study materials, providing diverse incentives, and addressing privacy concerns.

This report describes the study aims, design, and methods used to develop the CPOD infrastructure. Its innovative design facilitates comprehensive, multidisciplinary, and mechanistic translational research to elucidate biological and pathophysiologic linkages between structural racism, social determinants of health, and life experiences, and maternal, neonatal, and early childhood health outcomes. CPOD aims to address the critical need for more inclusive and representative perinatal research by prioritizing trust and engagement among diverse families within the Chicagoland area. While we primarily use the terms “maternal” and “mother” throughout this report to refer to the birthing parent, we recognize and appreciate that not all birthing people identify as female.

## Materials and methods

### Study aims and hypotheses

The CPOD study will test the overarching hypotheses that 1) specific social, environmental, and biological exposures in early pregnancy influence the risk of adverse maternal health and pregnancy outcomes, and 2) *in utero* social, environmental, and biological exposures influence early childhood physical and neurobehavioral development. Through a translational science approach with comprehensive, longitudinal, prospective survey and biospecimen data, we have established the key aims highlighted in **Table 1** to test these primary hypotheses using epigenetic, metabolomic, histopathologic, and immunologic methods.

**Table 1:** Key study aims.

AIM 1	Characterize environmental, social, and biologic exposures and health status during pregnancy, infancy, and early childhood in a diverse cohort of mother-fetus/infant dyads.	
**SUB-AIMS**	**1a.**	Determine transgenerational epigenetic markers of maternal adversity and trauma and evaluate associations with early childhood development.
	**1b.**	Evaluate the association of psychosocial stress, positive support structures, and social vulnerability with the metabolome in pregnancy and with maternal, neonatal, and early childhood outcomes.
	**1c.**	Identify placental markers of fetal growth restriction associated with early childhood growth and neurodevelopment.
	**1d.**	Determine the impact of microbiota composition of household members and pets on the development of the gut microbiome of young children.
	**1e.**	Evaluate the association of household dust and environmental chemicals (e.g., lead, quaternary ammonium compounds), in the context of housing and cleaning practices, with i) detection in human biospecimens, ii) infant microbiota/resistome, and iii) early childhood wheezing.
**AIM 2**	Develop a longitudinal biorepository of clinical data, patient-reported data, biospecimens, and environmental samples for future study in a diverse cohort of mother-fetus/infant dyads.	

### Participants, recruitment, and setting

A cohort of over 400 mother-fetus/infant dyads will be recruited from outpatient general obstetrics and maternal-fetal medicine clinics at two ambulatory sites in Chicago, Illinois: 1) Northwestern Medical Group (NMG), the primary faculty, fellow, and resident physician practice for Northwestern Medicine, and 2) Erie Family Health Center (Erie), a Federally Qualified Health Center with 11 community sites. The aim is to enroll a cohort of pregnant people in which a majority self-identify with a race other than White and/or self-identify as being of Latinx ethnicity, and with mixed socioeconomic backgrounds, including low-income and medically underserved individuals. All enrolled individuals plan to deliver at Northwestern Memorial Hospital (NMH), an urban academic quaternary care center partnered with Ann and Robert H. Lurie Children’s Hospital (Lurie). NMG and Erie provide care to racially, ethnically, and socioeconomically diverse pregnant people from the Chicagoland community. NMG provides care to both publicly and privately insured individuals, with demographics that mirror the epidemiology across Chicagoland. Erie provides care to publicly insured, underinsured, and uninsured individuals, and the majority of individuals who seek care at Erie self-identify as being of Latinx ethnicity.

**Fig 1:**
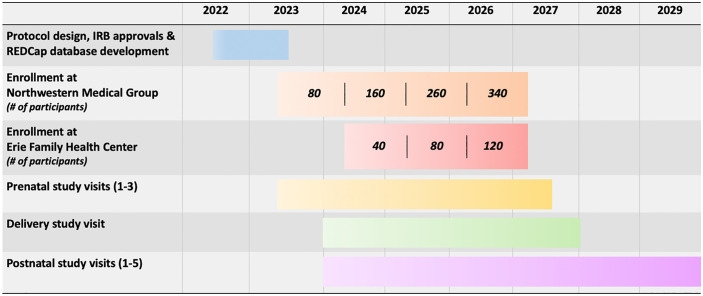
Overview of study timeline.

The Institutional Review Boards from Lurie and NMH approved the CPOD study protocol (IRB 2022–5510 and STU00218599, respectively); our study was further approved by Erie Family Health’s Research Ethics Committee. Recruitment was initiated in May 2023 in the NMG obstetric practices (Monday-Friday), and in August 2024 at Erie (one day per week due to site restrictions). We recruit approximately 80–90 participants at NMG annually, understanding NMG is a recruitment site for multiple competing large NIH-funded prospective and multi-site research studies and networks (e.g., Environmental Influences on Child Health Outcomes [ECHO], Maternal-Fetal Medicine Units [MFMU] Network, HEALthy Brain and Child Development [HBCD] Study, among others), in which participants cannot be co-enrolled into CPOD, in addition to 40 participants at Erie annually. Recruitment is anticipated to continue through 2027, with children followed postnatally through 2029 (**Fig 1**).

Pregnant people who obtain prenatal care at either site and plan to deliver at NMH are screened for eligibility at the time of their first prenatal visit. Individuals are eligible for inclusion if they have a viable singleton gestation, are between 8 weeks + 0 days (8 + 0 weeks) and 28 + 6 weeks gestation based on standard pregnancy dating criteria per the American College of Obstetricians and Gynecologists (ACOG), are 18 years of age or older, and speak English or Spanish [14]. Exclusion criteria include individuals who are incarcerated, living with HIV (due to preferential enrollment in HIV-specific cohort studies), cognitively unable to provide consent, plan to terminate the pregnancy, have a likely lethal fetal anomaly evident before enrollment, have enrolled in an intervention study anticipated to influence maternal or fetal outcomes, or are enrolled in another prospective study in which co-enrollment would be excessively burdensome for the participant.

Trained study personnel approach potentially eligible patients in-person during their routine prenatal care visits, confirm eligibility, explain the study objective and procedures in the participant’s native language and using a visual aid, obtain informed consent from interested individuals, and initiate study activities. If participants desire additional time to consider their options before enrolling (e.g., desire to discuss with their partner at home first), they are reapproached by research study staff and enrolled at their subsequent prenatal visit. The co-principal investigators contact potential study participants when indicated to address individual concerns or clarifying questions about the study protocol to further support enrollment. After enrollment, if a fetus or infant is subsequently identified to have a serious medical condition, they are not excluded from the study unless the family wishes to withdraw. Other adults or children living in the same household as the enrolled mother-fetus/child dyad are provided the opportunity for study participation with additional informed consent.

### Overview of CPOD study activities

**Fig 2:**
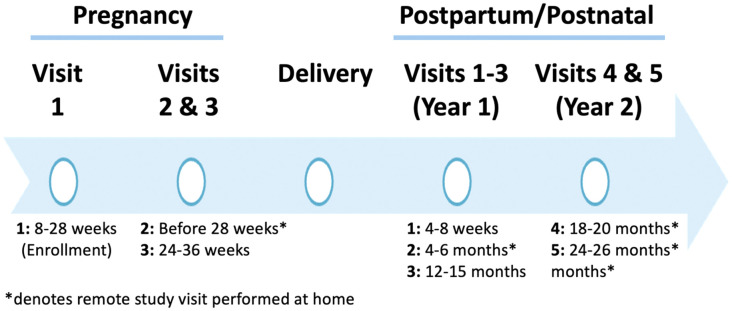
Timeline of study visits.

The CPOD study consists of nine study visits (**Fig 2**) and includes both in-person and remote activities. Study activities encompass self-reported questionnaires, semi-structured interviews, and biologic and environmental sample collections. Three study visits occur during pregnancy: 1) an in-person enrollment visit between 8 + 0 weeks and 28 + 6 weeks gestation with surveys and biospecimen collection, 2) a remote study visit with surveys and environmental sample collection at home completed anytime between enrollment through 28 + 6 weeks gestation, and 3) a third visit in person between 24 + 0 and 36 + 6 weeks gestation (ideally at least 8 weeks after enrollment) with surveys, a semi-structured interview, and biospecimen collection. Prenatal time points are paired with routine prenatal care visits, allowing longitudinal collection of biospecimens, health data, and self-reported social influences data with reduced logistical burden on participants. The fourth study visit is the delivery hospitalization, which involves only biospecimen collection.

Postnatally, there are five total visits when the baby is 4–8 weeks, 4–6 months, 12–15 months, 18–20 months, and 24–26 months. Postnatal visit activities include additional biospecimen collection from enrolled mothers and children, environmental specimen collections from participants’ homes, self-reported surveys, semi-structured interviews at two timepoints, and infant developmental assessments. Postnatal time points are predominately remote, spaced intentionally to minimize participant burden, and correspond to critical developmental milestones. The 4–6-month postnatal visit also includes biospecimen collection from consented household members and pets. Participants can complete most postnatal activities remotely, including all surveys (which can be completed at the participants’ own pace), some interviews, and most specimen collections. Only the 12–15-month postnatal visit is planned to occur in person, during which research staff will administer child neurodevelopmental assessments, and obtain biospecimens from both the infant and mother. Study visits last between 30–60 minutes, with only the 12–15 month visit requiring a longer in-person assessment (approximately 3 hours).

Accessible resources, incentives, and collaborative partnerships support enrollment, adherence, and retention and were informed by parental insight from the pilot study [13]. Monetary compensation is provided for participants’ time and effort, and transportation support is provided for in-person visits during the study period. Pertinent study results and clinically actionable findings that may adversely affect health of the enrolled children, mothers, and family members (e.g., abnormal child neurodevelopmental assessment results) are returned to participants along with referrals or resources, as needed (S1 Appendix).

### Survey instruments

At enrollment, sociodemographic characteristics, including partner relationship information and household composition, including other children and pets, are surveyed. Obstetric, medical, and medication history are collected by direct electronic record query. Infant medical conditions, medications, and developmental concerns are self-reported at multiple timepoints (**Table 2**).

**Table 2:** Surveys and questionnaires administered by study visit.

		Pregnancy visits			Postpartum/Postnatal visits				
					*Year 1*			*Year 2*	
Domains	Surveys and questionnaires	1	2	3	1	2	3	4	5
***Self-reported social and medical history***	Sociodemographic characteristics of the mother, partner (if applicable), other household members, and pets	X							
	Mother’s obstetric, medical, and medication history	X		X					
	Medical, medication, and developmental history of enrolled infant and other children in the household	X			X	X	X	X	X
***Environmental***	Household Cleaning Practices Questionnaire^a^		X			X		X	
***Nutrition***	National Cancer Institute Quick Food Scan [15]		X			X		X	
	Infant Feeding & Breast/chest-feeding Questionnaire [16]			X		X		X	
***Experiences of*** ***Discrimination***	Everyday Discrimination Scale (EDS) [17]		X						
	General Ethnic Discrimination (GED) Scale [18]						X		
***Social*** ***Vulnerability***	Accountable Health Communities Health-Related Social Needs Screening Tool (AHC-HRSN) [19]		X				X		
	Structural Vulnerability Assessment Tool (SVAT) [20]			X					
	Trauma and Life Experiences Semi-Structured Interview^b^			X	X			X	
***Social support***	Multi-Dimensional Scale of Perceived Social Support [21]		X				X		
	Adapted Postpartum Social Support Questionnaire [22]				X				
***Psychosocial stress***	Perceived Stress Scale (PSS) [23]		X				X		
	Prenatal Distress Questionnaire [24,25]			X					
	Parental Stress Scale [26]				X				
	Postpartum Childcare Stress Questionnaire [27]				X				
***Mental health***	Patient Health Questionnaire-9 (PHQ-9, depression screen) [28]	X		X	X				
	Generalized Anxiety Disorder-7 (GAD-7) [29]	X		X	X				

^a^See S2 Appendix.

^b^See S3 Appendix (modified from the previously validated Psychosocial Assessment Screening Tool [30,31]).

Standardized and validated survey instruments assess nutrition, experiences of discrimination and racism, social vulnerability, support systems, psychosocial stress, and mental health during pregnancy and postpartum (**Table 2**). A household cleaning practices survey was also developed based on literature regarding quaternary ammonium compound exposure (S1 Appendix 2) [32]. Intergenerational childhood adversity, trauma, and life experiences are assessed using a semi-structured interview during pregnancy, 6 weeks postpartum, and 18 months postpartum. Interviews are conducted in-person or via phone or secure Zoom, audio-recorded, and transcribed. The trauma and life experiences interview guide, modified from the validated Psychosocial Assessment Tool, covers topics including abuse history, household disciplinary practices, and parental feelings toward their child (S3 Appendix) [30,31,33].

### Postnatal infant developmental assessments

Standardized, validated measures assess early childhood neurodevelopment, language, communication, and behavior at five time points over the first two years of life (**Table 3**, S4 Appendix). Assessments are longitudinal and age-appropriate, with corrected postnatal age used as indicated. Most activities are performed remotely, except for in-person assessments at 12–15 months.

**Table 3:** Infant developmental questionnaires and assessments.

	Questionnaires and assessments	Postnatal visits					
		* Year 1*			* Year 2*		
		1	2	3		4	5
***Development***	Ages and Stages Questionnaires, Third Edition (ASQ®-3) [34]	X	X			X	X
	Bayley Scales of Infant and Toddler Development, Fourth Edition (Bayley™-4) [35]			X^a^			
	Modified Checklist for Autism in Toddlers, Revised with Follow-up (M-CHAT-R/F) [36]					X	
***Language and Communication*** ^b^	Preschool Language Scales, Fifth Edition (PLS™-5) [37]			X			
	MacArthur-Bates Communicative Development Inventories (MCDI) Words and Sentences short form [38]						X
***Behavior***	Multidimensional Assessment Profiles Temper Loss Scale (MAPS-TL) [39,40]	X	X			X	
	Early Regulation in Context Assessment with Family Culture Matters Activity (ERICA-FCM) [41]			X^a^			X

^a^The Bayley™-4 assessment and ERICA-FCM at 12 months is completed in person. All other infant assessments are performed remotely.

^b^The ASQ®-3 and Bayley™-4 also include domains assessing language and communication.

### Biospecimen collection

**Table 4** summarizes biologic and environmental specimens collected by study visit. Sample collection, processing, and storage methods are detailed in S5 Appendix. Aliquots are banked for future research. Urine is stored for metabolomic assays and/or other future analyses. Plasma is stored for future metabolomic analyses and inflammatory cytokine assays. PBMCs are stored for immunologic analysis. Whole blood processed with Zymo DNA/RNA™ shield solution and buccal swabs are stored for planned epigenetic analyses. Placental samples are stored for metabolomic analyses, and glass slides prepared for histologic interpretation by a trained perinatal pathologist. Salivary, nasal, vaginal, and rectal swabs are collected for microbial analysis. Infant and pet stool samples will also undergo microbial analysis. Future analyses of tap water include measurement of lead and/or other heavy metals, quaternary ammonium compounds, and other chemicals from household cleaning products. Dust samples will be used to assess contamination from household cleaning products (e.g., quaternary ammonium compounds).

**Table 4:** Biologic and environmental specimen collections by study visit.

Biologic and environmental specimens	Pregnancy visits			Delivery hospitalization	Postpartum/ Postnatal visits				
					Year 1			Year 2	
	1	2	3		1	2	3	4	5
***Maternal biospecimens, during pregnancy***									
Urine (20mL)	X		X						
Blood (5-10mL), plasma & PBMCs	X		X	X					
Blood (3mL), whole	X		X	X					
Buccal swab (1 Sk-1 swab with Simhelix Dri-Capsule, self-collected)				X			X		
Saliva (1 oral FLOQ® swab, self-collected)	X		X			X		X	
Vaginal swab (1 swab, self-collected from mid-vagina)^b^	X		X			X		X	
Rectal swab (1 swab, self-collected)^b,c^	X		X			X		X	
***Delivery specimens***									
Placenta (2 cm^3^, divided into 3 samples), flash frozen				X					
Placenta (2 cm^3^, divided into 3 samples), processed with Zymo DNA/RNA™ shield solution				X					
Placenta (2 cm^3^, divided into 3 samples), formalin fixed				X					
Cord blood (5–10 ml), plasma				X					
Cord blood (5–10 ml), PBMCs				X					
Cord blood (3 ml), whole				X					
***Neonatal and early childhood biospecimens***									
Urine							X		
Blood^a^ (5-10mL), plasma & PBMCs							X		
Blood^a^ (3mL), whole							X		
Buccal swabs (1 Sk-1 swab with Simhelix Dri-Capsule, staff-collected)				X			X		
Saliva (1 oral FLOQ® swab)				X		X		X	
Nasal fluid (1 nasal FLOQ® swab)				X		X		X	
Stool sample (non-meconium)				X		X		X	
***Biospecimens from other household member(s) (siblings, other household adults)***									
Buccal swab (1 Sk-1 swab with Simhelix Dri-Capsule, self-collected)						X			
Saliva (1 oral FLOQ® swab, self-collected)						X			
Nasal fluid (1 nasal FLOQ® swab)						X			
Rectal swab (1 swab, self-collected)^b,c^						X			
***Environmental samples***									
Household dust, from vacuum		X				X		X	
Household tap water						X			
Pet stool sample (if applicable)						X			

*PBMCs—peripheral blood mononuclear cells*

*Detailed collection and processing protocols for all biologic and environmental specimens are available upon request.*

^a^If unable to be obtained by a trained research phlebotomist, then a blood sample (500 microliters total) will be collected from the child via supervised application of the TAP® Micro Select capillary blood self-collection device [42].

^b^If participant was not comfortable with self-collection, then vaginal and/or rectal swab was collected by a clinical provider.

^c^If participant declined rectal swab, then stool sample was collected where able.

### Pilot analyses for validation of participant biologic and environmental sample collection

For both self-collected biologic and environmental samples and biologic samples collected by research staff, we performed several pilot analyses on 5−10 samples collected early in the study implementation phase to refine our study protocol and ensure appropriate sample collection, storage methods, and yield of the biologic marker of interest. Specifically, we compared concentrations of PBMCs isolated when blood was stored at room temperature versus refrigerated at −4°C, prior to processing for long term storage at -80^o^C, and identified higher yield of PBMCs isolated when blood samples were stored at room temperature; thus, for PBMC isolation, blood samples were stored at room temperature prior to PBMC isolation.

Given the intended use of buccal swabs for epigenetic analyses, we compared yield of DNA from self-collected and research staff-collected buccal swabs, and found adequate concentrations of DNA from both adults and children. We also ensured adequate DNA and RNA concentrations from whole blood collected in Zymo DNA/RNA™ shield solution (Zymo Research Corporation, Irvine, CA). Pilot analyses of vaginal and rectal microbiome swabs were not performed, given abundant evidence demonstrating similar efficacy of provider- and patient-collected vaginal and rectal swabs for microbiome analyses, and similar efficacy of stool and rectal swab collection for evaluation of the gut microbiome [43–50]. Finally, we performed pilot analyses on home dust samples to validate our dust collection and processing methods for detection of benzalkonium chloride, a quaternary ammonium compound, via high-performance liquid chromatography-mass spectrometry.

### Outcomes and measures

#### Obstetric outcomes.

The primary composite obstetric outcome of the CPOD cohort is defined as having at least one of the following placentally-mediated adverse pregnancy outcomes: hypertensive disorders of pregnancy (HDP), preterm birth (PTB), fetal growth restriction (FGR), and/or intrauterine fetal demise (IUFD). We will further evaluate HDP, PTB, FGR and IUFD individually as secondary outcomes, among additional obstetric outcomes (**Table 5**).

**Table 5:** Obstetric, neonatal, and early childhood outcomes.

Outcome	Definition
***Obstetric outcomes***	
Composite adverse pregnancy outcome	Composite of placentally-mediated adverse outcomes: HDP, PTB, FGR, and IUFD
Hypertensive disorders of pregnancy (HDP)	Gestational hypertension, preeclampsia with or without severe features, chronic hypertension with superimposed preeclampsia with or without severe features, eclampsia, and HELLP syndrome (hemolysis, elevated liver enzymes, and low platelet count) based on standard definitions per ACOG [51]
Preterm birth (PTB)^a,b^	Delivery of a liveborn or stillborn infant for any cause between 20 weeks + 0 days and 36 + 6 weeks gestation; *very PTB* (birth between 20 + 0 and 33 + 6 weeks gestation) and *spontaneous* and *medically indicated* PTB will also be assessed
Fetal growth restriction (FGR)	Estimated fetal weight (EFW) and/or abdominal circumference (AC) less than the 10^th^ percentile on antenatal ultrasound, per the Hadlock growth curve; *severe FGR*, defined as EFW and/or AC less than the 3^rd^ percentile on antenatal ultrasound, will also be assessed [52]
Intrauterine fetal demise (IUFD)	Absent fetal cardiac activity at ≥20 + 0 weeks’ gestation confirmed on ultrasound
*Additional obstetric outcomes*	Cesarean birth, operative vaginal birth, 3^rd^ or 4^th^ degree laceration, need for blood product transfusion, intraamniotic infection requiring antibiotics, postpartum endometritis, wound infection, venous thromboembolism, peripartum hysterectomy or other unanticipated intrapartum or postpartum surgery, intensive care unit admission, or maternal death
***Neonatal outcomes***	
Composite neonatal morbidity	Respiratory support within 72 hours of birth, five-minute Apgar score of 3 or less, respiratory distress syndrome, bronchopulmonary dysplasia, persistent pulmonary hypertension, hypoxic ischemic encephalopathy, neonatal seizure, culture-proven neonatal sepsis or pneumonia, meconium aspiration syndrome, necrotizing enterocolitis, intracranial or subgaleal hemorrhage, shoulder dystocia, birth trauma (e.g., neonatal brachial plexus palsy, facial nerve palsy, retinal hemorrhage, or bone fracture), hypotension requiring vasopressor support, and neonatal death [53]
Small-for-gestational age^c^	Neonatal birthweight <10^th^ percentile
Large-for-gestational age^c^	Neonatal birthweight ≥90^th^ percentile
*Additional neonatal outcomes*	Hyperbilirubinemia requiring phototherapy or exchange transfusion, hypoglycemia requiring intravenous dextrose therapy, or neonatal intensive care unit admission
***Infancy and early childhood outcomes***	
Composite adverse childhood development^d,e^	Growth-faltering, malnutrition, gross motor delay, hearing impairment, vision impairment, speech delay, or infant or childhood death
Infant death	Death before 1 year of age
Childhood death	Death at ≥1 year of age
*Additional early childhood outcomes* ^*f*^	Cerebral palsy, autism spectrum disorder, behavioral dysregulation, chronic lung disease (including reactive airway disease/asthma), food or medication allergy

^a^*Spontaneous preterm birth (sPTB):* delivery prior to 37 + 0 weeks gestation that occurs secondary to spontaneous onset of preterm labor, preterm prelabor rupture of membranes, or fetal membrane prolapse [5]

^b^*Indicated preterm birth (iPTB)*: delivery prior to 37 + 0 weeks gestation that is medically recommended (and proceeds via induction of labor or cesarean birth) due to concern for potential compromise to maternal or fetal health, including HDP, FGR, placental abnormality (placenta previa, vasa previa, placenta accreta spectrum, placental abruption), intra-amniotic infection, maternal medical conditions, fetal surveillance with abnormal test results, and/or congenital anomalies warranting PTB.

^c^per the World Health Organization (WHO) Child Growth Standards for full-term infants and the Fenton Growth Chart for preterm infants

^d^*Growth-faltering:* weight-for-length or body mass index lower than expected, or weight loss greater than two major percentile lines after established steady growth trajectory on comparable age- and sex-specific growth charts [54]

^e^*Malnutrition:* stunting (height-for-age < −2 standard deviations [SD]), wasting (weight-for-age < −2 SD), overweight (weight-for-height > +2SD), or underweight (weight-for-age < −2 SD), relative to the WHO Child Growth Standards median [55]

^f^confirmed by clinician diagnosis

#### Infant outcomes.

Infant outcomes include those in the neonatal (<28 days of life), infancy (1–11 months), and early childhood (12–24 months) developmental periods. Composite neonatal morbidity is the primary neonatal outcome [53], defined as having at least one of the following: respiratory support within 72 hours of birth, five-minute Apgar score of 3 or less, respiratory distress syndrome, bronchopulmonary dysplasia, persistent pulmonary hypertension, hypoxic ischemic encephalopathy, neonatal seizure, culture-proven neonatal sepsis or pneumonia, meconium aspiration syndrome, necrotizing enterocolitis, intracranial or subgaleal hemorrhage, shoulder dystocia, birth trauma (e.g., neonatal brachial plexus palsy, facial nerve palsy, retinal hemorrhage, or bone fracture), hypotension requiring vasopressor support, and neonatal death. We will assess individual neonatal morbidities and other neonatal outcomes as secondary outcomes.

#### Early childhood outcomes.

The primary early childhood outcome is a composite adverse development outcome defined as having at least one of the following: growth-faltering, malnutrition, gross motor delay, hearing impairment, vision impairment, speech delay, and/or infant or childhood death by two years of life. Additional early childhood outcomes, confirmed by clinician diagnosis, are also outlined in **Table 5**. Use of a composite obstetric, neonatal, and early childhood outcome is clinically and scientifically appropriate given similar risk factors for the conditions included in each composite outcome.

### Data management

Trained study personnel will perform chart abstraction. The co-principal investigators complete quality control audits quarterly via re-abstraction on a random selection of charts with and without the primary obstetric and infant outcomes. Prenatal, delivery, and neonatal record abstraction are performed once both mother and child are discharged from their delivery hospitalization. Pediatric chart extraction will be performed at 24-months of life. S6 Appendix contains further detail regarding clinical data abstracted from the electronic health record.

Database audits are performed quarterly to assess completion rates of survey instruments, semi-structured interviews, and biospecimen collection. Strategies to improve fulfillment and fidelity of all study activities among participants are discussed to address any gaps. For the Trauma and Life Experiences Semi-Structured Interview, recordings of the interviews are audited every 3–6 months by a licensed social worker and collaborator with extensive experience performing interviews among trauma victims to provide feedback and re-training to individual research staff members, in order to enhance the qualitative data obtained and support provided to participants during these interviews.

Most data collected from CPOD participants are securely maintained via Northwestern University Feinberg School of Medicine’s (NUFSM) Research Electronic Data Capture (REDCap) database. Participant-reported survey data is directly captured via the REDCap platform. Clinical data extracted from the electronic health record or merged from NUFSM’s Electronic Data Warehouse (EDW) and/or the Chicago Area Patient Centered Outcomes Research Network (CAPriCORN) will also be stored in REDCap [56]. A subset of CPOD data is collected by research staff via direct query or interview (either in person or remotely via a secure Zoom platform) and stored on secure drives. Mother and infant records are linked using a unique CPOD identification number assigned to study participants. CAPriCORN houses data from adult and children’s hospitals, community health centers, primary care clinics, and outpatient specialty clinics throughout Chicago [56]. Merging CAPriCORN data with other sources of study data will promote the development of vital health information technology infrastructure to support future perinatal research in Chicago, particularly that utilizing the CPOD biorepository of data and specimens.

### Statistical considerations

CPOD has a target enrollment of over 400 maternal-infant dyads. Anticipating approximately 15% loss-to-follow up and/or withdrawals in this longitudinal cohort, we plan to recruit 460 participants to maintain an analytic sample of 400 participants. For the primary obstetric, newborn, and infant developmental outcomes, we have computed effect size estimates (i.e., odds ratios) for Pearson’s Chi-square tests with a 2-sided alpha of 0.05, estimating 5–20% of control subjects experience varied exposures (common exposure, 20%; rare exposure, ≤ 5%) [5]. Inverse probability weighting or multiple imputation, depending on the specific analysis, will be performed for missing data.

For obstetric outcomes, it is estimated this cohort will yield 40–60 cases of a HDP (10–15%), 40–60 cases of PTB (10–15%), 40 cases of FGR (10%), and 2–3 cases of stillbirth (0.6%), with approximately 25% exhibiting concurrence of two or more outcomes [57–60]. We assumed a 5% event rate for the primary neonatal outcome, or a sample size of 25 cases, derived from the rate of composite neonatal morbidity (4.3–5.4%) in a multicenter trial of low-risk nulliparas [53]. Finally, for the primary early childhood developmental outcome, national estimates of adverse early childhood development are 5.9–6.9%, reflective of the proportion of 2 year-old children served by the Individuals with Disabilities Education Act as of 2018 [61]. We thus assumed a 6% event rate for the primary early childhood outcome, or a sample size of 30 cases. The effect size calculations in **Table 6** are illustrative, and we recognize that power needed will vary based on each of our aims and sub-aims. Specific analytic plans and effective size calculations for each sub-aim are detailed in S7 Appendix.

**Table 6:** Power calculation.

	Estimated sample size *(N cases)*	Exposure rate in controls	Estimated power	Estimated effect size (Odds Ratio)
Composite adverse pregnancy outcome	100	20%	>80%	1.6
		5%		2.4
Composite neonatal morbidity	20	20%	>80%	1.5
		5%		2.0
Composite adverse childhood development	25	20%	>80%	1.5
		5%		2.0

We will employ standard parametric versus nonparametric methods and asymptotic versus exact methods for descriptive and inferential analyses to address our aims. We will use binary, ordinal, and multinomial variables to describe characteristics of the cohort and nested cohorts, estimate effects, identify associations between exposures and outcomes, and inform stratification and modeling adjustments for confounders and effect modifiers. Continuous and longitudinal models will test key hypotheses and identify effect modifiers of identified associations. Employing fit regression models, we will determine associations between maternal exposures and obstetric and child outcomes, and examine potential mediators via structural equation modeling, accounting for covariates that may differ depending on the outcome. All statistical analyses will be conducted using STATA, R, and Python [62].

### Analysis of the trauma and life experiences semi-structured interview

Using methods previously described by our team [63], the research team developed an initial codebook deductively based on pre-existing knowledge and the *Trauma and Life Experiences Semi-Structured Interview* guide, after which each individual member of the coding team coded the same five transcripts. The coding team met to establish agreement, ensure reliability, and refine code definitions in the finalized codebook. All subsequent transcripts are primary and secondary coded and disagreements are resolved during research team discussions. Three team members review code excerpts to identify themes, and each piece of raw datum is assigned to appropriate categories. To account for ongoing data collection with interim analyses, the research team continues to refine the codebook, comparing coding approaches and adjusting definitions or creating new codes, categories, and themes as they emerge. We aim to qualitatively describe experiences of trauma and life experiences of pregnant people, associate experiences with epigenetic changes in the pregnant person, and identify if these epigenetic changes are passed transgenerationally to offspring by correlating with epigenetic analyses of paired maternal-child buccal swabs.

## Discussion

As a large cohort of racially, ethnically, and socioeconomically diverse pregnant people (**Table 7**) and their infants with in-depth data, CPOD will allow for improved understanding of the linkage between social and environmental exposures in pregnancy with adverse obstetric outcomes and early childhood health and development. The CPOD study and repository will allow correlative analyses and translational studies that can uncover biological mechanisms that underlie adverse outcomes. Several prior studies have attempted to connect pathophysiological processes in perinatal health and psychosocial stress with social determinants of health and racial/ethnic disparities [10,64–68]. However, most studies focus on stress pathways, whereas additional microbiologic, immunologic, metabolic, environmental, and epigenetic, among other, mechanisms may be involved [69–74]. This body of literature is limited in both quantity and quality at present, with few studies able to garner a comprehensive social, environmental, and biologic dataset linked from pregnancy through childhood. The rich and comprehensive data acquired through CPOD will further advance our knowledge of these intergenerational mechanisms among a diverse population.

**Table 7:** Sociodemographic characteristics of enrolled participants.

*Characteristics*	Overall *median (IQR)* *or n (%)* N = 214	NMG *median (IQR)* *or n (%)* n (%), N = 184	Erie *median (IQR)* *or n (%)* n (%), N = 30
Maternal age, years	32 (29, 35)	32 (29, 36)	30 (26, 32)
Gestational age at enrollment, weeks	20 (16, 24)	20 (16, 20)	21 (14, 26)
Race and Ethnicity American Indian or Alaskan Native Asian Non-Hispanic Black White Other Decline to respond	8 (3.7) 20 (9.4) 46 (21.5) 94 (43.9) 39 (18.2) 7 (3.3)	5 (2.7) 19 (10.4) 46 (25.0) 88 (47.8) 26 (14.1) 0 (0.0)	3 (10.0) 1 (3.3) 0 (0.0) 6 (20.0) 13 (43.4) 7 (23.3)
Hispanic/Latinx ethnicity Declined to respond Insurance Private insurance Public insurance Uninsured Other	71 (33.2) 1 (0.5) 128 (59.8) 80 (37.4) 4 (18.7) 2 (0.5)	43 (23.4) 1 (0.5) 125 (67.9) 56 (30.5) 2 (1.1) 1 (0.5)	28 (93.3) 0 (0.0) 3 (10.0) 24 (80.0) 2 (6.7) 1 (3.3)
Income < $35,000 $35,000-100,000 > $100,000 Decline to answer	53 (24.8) 48 (22.4) 107 (50.0) 6 (2.8)	34 (18.5) 38 (20.7) 107 (58.1) 5 (2.7)	19 (63.4) 10 (33.3) 0 (0.0) 1 (3.3)

*NMG—Northwesern Medical Group, Erie—Erie Family Health Center*

### Strengths and limitations

The CPOD cohort study has several notable strengths. The multidisciplinary CPOD study team comprises experts from myriad specialties committed to executing the study protocol with scientific rigor. The prospective and data-intense nature of the protocol, with broad biospecimen collection from early pregnancy through two years of postnatal life, provides the opportunity for a wealth of demographic, psychosocial, environmental, and biomarker data to be incorporated into a broad range of mechanistic scientific analyses underlying maternal and child health outcomes. Using several translational science approaches and a variety of biospecimen analyses (e.g., epigenetics, metabolomics, placental histopathology, microbiome analyses) to address key study aims, data from this cohort will provide clinical and mechanistic insights into how environmental and psychosocial exposures, both during pregnancy and transgenerationally, influence changes in the underlying biology of maternal-child dyads, and how these changes are associated with the risk of adverse health outcomes that contribute to future disease.

In both pre-defined and exploratory analyses, CPOD’s rich mixed-method repository of data will allow us to address novel questions evaluating the biologic linkage of psychosocial and environmental exposures with pregnancy outcomes and childhood development via a comprehensive translational research approach. Additionally, the CPOD biorepository provides investigators the opportunity to perform multiple ancillary and pilot studies with greater agility compared to other national cohorts with similar coalition design, such as the Environmental influences on Child Health Outcomes (ECHO) program. Findings from these ancillary studies may then be validated in collaboration with national cohorts such as ECHO. Further, the emphasis on diverse populations and participatory-informed design of the CPOD cohort is ideally poised to improve our understanding of heterogeneous phenotypes of adverse pregnancy outcomes and childhood developmental disorders; provide valuable insights into risk and protective factors of maternal, neonatal, and early childhood morbidity; and identify novel diagnostic and therapeutic targets that we may harness to improve care among diverse populations during pregnancy and postnatally.

Beyond the data-rich and interdisciplinary nature of the CPOD study, the longitudinal follow-up with repeated measures during pregnancy and postnatally enhances our ability to phenotype and assess changes over time. Serial collection of social, environmental, and biologic data may further yield important mechanistic insights into maternal and child health outcomes. We utilize validated questionnaires, neurodevelopmental assessments, and novel data collection tools, such as a semi-structured interview guide and adapted assessments, which are informed by our interdisciplinary team of co-investigators and content experts, to obtain high-quality longitudinal data on psychosocial exposures and childhood development.

Logistically, we have focused recruitment at two high-volume prenatal care centers, linked to one delivery hospital. Our research team is available 24/7 for around the clock coverage, particularly for unscheduled deliveries outside of business hours, allowing for timely biospecimen sample collection, processing, and storage of time-sensitive samples (e.g., plasma, placenta, cord blood). Incorporation of remote participation for a majority of study activities, particularly during the postnatal period (e.g., surveys, swabs, dust, and water collection), reduces participant burden while enhancing study feasibility and participant retention throughout this longitudinal study.

Finally, a socioeconomically and racially diverse population from throughout the Chicagoland community comprises the CPOD cohort. This cohort is enriched with individuals and families who identify as non-White and Latinx who have been historically underrepresented in perinatal research. The diverse composition of the CPOD cohort will enhance the generalizability of the study findings to other urban and metropolitan communities throughout the U.S., and we anticipate these findings will be externally validated across other national cohorts such as ECHO. Notably, CPOD was designed with community stakeholder input from these aforementioned underrepresented groups, with ongoing implementation feedback from a Participant Advisory Board. Clinically actionable study results from this cohort will be directly returned to individuals and their communities, which may not be feasible with similar multicenter cohorts. CPOD thus exemplifies a unique commitment to community representation and engagement in research.

Although CPOD has several strengths, we must recognize its limitations. The sample size may limit our ability to evaluate rare maternal and child health outcomes or perform predictive modeling for adverse outcomes. However, we anticipate the ability to demonstrate variation across the spectrum of both normal and abnormal pregnancy and early childhood life courses. Although we will not exclude preterm infants in postnatal follow up, we may be underpowered to evaluate developmental outcomes associated with prematurity in this subgroup. Overall, we recognize that with the expected sample size in this cohort, the possibility of type I error remains for significant findings identified in each of our study aims, and external replication of our results will remain necessary.

Although incorporation of remote study activities has the potential to enhance participant engagement and retention longitudinally, we do anticipate some degree of loss-to-follow up and missing data, including among the additional household members we seek to enroll. We will attempt to mitigate this risk of attrition bias through a multipronged approach: 1) establishing strong rapport with participants in-person at the initial visit, follow-up prenatal visits, at delivery, and the 12-month postnatal visit, 2) employing frequent and personalized communication with participants (e.g., timely reminders of upcoming study activities, birthday cards mailed from the study team for enrolled infants), 3) incentivizing participants for ongoing participation and completion of study activities by increasing compensation for each subsequent study visit, 4) making follow-up activities as brief, flexible, and convenient for participants as possible, including flexing in-person visits to remote visits when indicated, and 5) returning results of childhood developmental assessments and other clinically actionable measures to enrolled birthing parents (S1 Appendix). As previously described, we will also employ appropriate statistical methods (i.e., inverse probability weighting or multiple imputation, depending on the specific analysis) to address missing data. We are encouraged that we will be able to minimize attrition, as our clinical site has a reputation for successfully maintaining high participant retention greater than 90%, protocol adherence, and data quality in other similar longitudinal studies performed at our site over the past two decades, as a result of our well-established clinical research structure to support prospective obstetric, neonatal, and pediatric cohort studies. At the time of this publication, the study has met approximately 50% of its enrollment goal, and retained more than 94% of study participants through delivery to postnatal follow up.

Finally, while we have made eligibility criteria intentionally broad to reduce selection bias, with any longitudinal study enrolling antenatally, there is a potential for selection bias by selecting individuals who are engaged in prenatal care and willing to participate in research. For all patients screened for eligibility but not enrolled, we record basic sociodemographic information (i.e., maternal age, race, ethnicity, primary language), and reasons why they were not enrolled (e.g., missed clinic visit, priority for enrollment in alternate study, declined due to privacy concerns or mistrust of research, etc.) in the REDCap database to be able to characterize selection bias related to inclusion into the study in future analyses and manuscripts produced from this cohort.

### Future directions and conclusions

The CPOD study will serve as a platform for future ancillary studies, beyond the current study aims, focused on evaluating mechanisms underlying perinatal health outcomes through a health equity lens. Discovery of early pregnancy factors and high-risk and protective social and environmental exposures among different racial, ethnic and economically resourced groups that contribute to adverse pregnancy outcomes and abnormal childhood development may reveal significant opportunities for public health impact. This study may identify targets for prevention and facilitate earlier intervention among key subgroups identified through this research initiative. CPOD’s engagement of community members, diverse multidisciplinary team of collaborators, and commitment to inclusivity and representation of the greater Chicago community in the process of study development and implementation places historically marginalized individuals and their families at the center of this translational research collaboration. Translating the findings from the CPOD cohort that link adverse social and environmental exposures with clinical outcomes during a transformative time in the lives of parents and their offspring has the potential to reveal important opportunities for reduction of maternal and child health disparities.

## Supporting information

S1 Appendix Returning of results to participants. (DOCX)

S2 Appendix Household Cleaning Practices Questionnaire. (DOCX)

S3 Appendix Trauma and Life Experiences Semi-Structured Interview. (DOCX)

S4 Appendix Postnatal infant developmental assessments. (DOCX)

S5 Appendix Biologic and environmental specimen collection, processing, and storage. (DOCX)

S6 Appendix Chart abstraction variables. (DOCX)

S7 Appendix Analysis plan for key study aims/sub-aims. (DOCX)
