# Supplementary material for: The Founders’ 400 and Chicago Perinatal Origins of Disease study protocol: Following a prospective, longitudinal cohort from early pregnancy through two years of postnatal life
Source: PLoS One. 2025 Sep 29;20(9):e0332928. doi: 10.1371/journal.pone.0332928 (PMC12478913; doi:10.1371/journal.pone.0332928)
Supplement: S1 Appendix — (DOCX) [file pone.0332928.s001.docx]

**Appendix 1. Returning of results to participants**

Results of several infant neurodevelopmental and environmental assessments are returned to enrolled birthing parents throughout the course of the study, as outlined in S1 Table below. Additionally, when high PHQ-9 and/or GAD-7 scores (≥10) [1, 2] and/or built environment hardships are identified on other survey assessments or during the semi-structured interviews, referrals to mental health support, a licensed clinical social worker, and/or other referrals to community resources are provided to enrolled participants. Clinical providers are also notified of high PHQ-9 and/or GAD-7 scores among enrolled participants to inform patient care.

**S1 Table. Results returned at study visits**

| **Visit** | **Pregnancy Visits** | **Postnatal Visit 1**  (4-8 weeks) | **Postnatal Visit 2**  (4-6 months) | **Postnatal Visit 3**  (12 months) | **Postnatal**  **Visit 4**  (18 months) | **Postnatal Visit 5**  (24 months) |
| --- | --- | --- | --- | --- | --- | --- |
| Neurodevelopmental  assessments |  | ASQ®-3,  2-month | ASQ®-3,  4- or 6-month | ASQ®-3,  12-, 14-, or 16-month  PLS™-5  **Bayley™-4**^1^ | ASQ®-3, 18- or 12-month  M-CHAT-R/F | ASQ®-3,  24-month |
| Environmental exposures |  |  | Lead in household water |  |  |  |
|  | QAC levels in dust |  | QAC levels in dust |  | QAC levels in dust |  |
| Mental health | PHQ-9, GAD-7 | PHQ-9,  GAD-7 |  |  |  |  |
| Built environment | Built environment hardships^2^ (food, housing, safety concerns, etc.) | | | | | |

*ASQ®-3—****Ages and Stages Questionnaires, Third Edition; Preschool Language Scales, Fifth Edition (****PLS™-5),* ***Bayley****™****-4—****Bayley Scales of Infant and Toddler Development, Fourth Edition;* ***M-CHAT-R****/F—Modified Checklist for Autism in Toddlers, Revised; QAC—quaternary ammonium compound; PHQ-9—Patient Depression Questionnaire-9; GAD-7—General Anxiety Disorder-7*

^1^Includes the in-person assessment of the ***Bayley***™***-4*** Cognitive and Motor components, or the remote assessment of the ***Bayley***™***-4*** Social-Emotional and Adaptive Behavior Survey.

^2^Built environment hardships are assessed at various timepoints throughout the study through survey assessments (Accountable Health Communities Health-Related Social Needs Screening Tool [AHC-HSRN], pregnancy visit 2 and postnatal visit 3; Structural Vulnerability Assessment Tool [SVAT], pregnancy visit 3) and semi-structured interviews (pregnancy visit 3; postnatal visits 1 and 4).

***Example Return of Results Report Template for Postnatal Visit 3***

Dear [Adult Participant’s Name],

Thank you for your participation in the Chicago Perinatal Origins of Disease (CPOD) study at Lurie Children’s Hospital! We are so thankful for the time and effort you and [baby name] have contributed to our study.

Below is a report of information we learned about your child at their third study visit. This report includes descriptions of the tests and the score report. **These results are meant for research purposes only, and cannot be used to identify a disease or an issue with development.** If you have any questions about the score report, tests, or study please do not hesitate to contact us by email at lmithal@luriechildrens.org; tnbranche@luriechildrens.org. We look forward to keeping in touch with you and your family, and thank you again for your participation!

Sincerely,

**The CPOD Team**

**Lurie Children’s Hospital & Northwestern Medicine**

**Child’s name:** **Age at assessment (in months):**

**Summary:** As a part of your recent CPOD study visit on [date of assessment] your child completed the following evaluation:

- The Bayley Scales of Infant and Toddler Development, Fourth Edition (Bayley™-4)
- Preschool Language Scales, Fifth Edition (PLS™-5)

**About the Bayley Scales of Infant and Toddler Development™ (Bayley™-4)**

The Bayley 4 examines 5 areas of development:

- Gross motor skills (how your child moves their body)
- Fine motor skills (how your child uses their hands)
- Expressive language (how your child speaks about things)
- Receptive language (your child’s understanding of language)
- Cognitive skills (how your child thinks about things and solves problems)

Scores on the Bayley™-4 provide a snapshot of your child’s current development as observed

during the testing session. A child's scores may be influenced by their motivation, attention,

interests, and energy-level that day, and a test score cannot tell all the skills that your child

might be able to use. It is also important to remember that your child was seen in an unfamiliar place with new people and different toys, so their behavior may have been different from what

you see at home.

Because the Bayley™-4 was administered as part of a research study by our research staff, it is

not the same as having your child’s doctor perform a test. However, if your child receives a

result of “Below Expected”, we suggest that you follow-up with [CHILD’S NAME] health care

provider regarding your child's development. You can also call 1-800-843-6154 (Early

Intervention), which is a state-wide number that coordinates services, including developmental

evaluations of babies. Our study team can also help you find services to get more testing done.


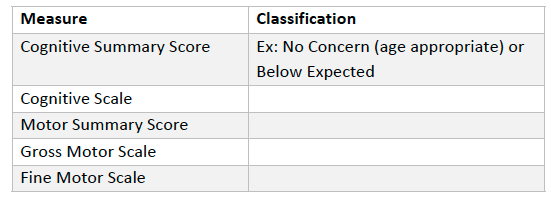


**About the Preschool Language Scales Fifth Edition™ (PLS™-5)**

Preschool Language Scales, Fifth Edition (PLS™-5) assesses your child’s language development by both observing the language they use during their in-person visit and interacting with them.

Some items are also scored based on your own parent-report of their day-to-day skills. Scores

on the PLS™-5 will provide a snapshot of your child’s language as observed during your visit.

Again, your child's scores may be influenced by their motivation, attention, interests, and

energy-level that day. The PLS™-5 was administered as part of your research visit and cannot be

used to official diagnose a language delay; however, if your child receives a result of “Below

Expected”, we suggest that you follow-up with {CHILD’S NAME} health care provider regarding

your child's development.


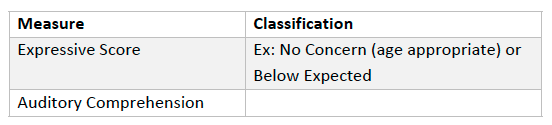


**Additional Resources**

If you are interested in learning more about cognitive, motor, and language development, as well as what developmental stages are next for your little one, we recommend checking out these resources from outside Lurie Children’s Hospital.

[**American Academy of Pediatrics: Ages and Stages Toddler**](https://www.healthychildren.org/English/ages-stages/toddler/Pages/default.aspx)

*https://www.healthychildren.org/English/ages-stages/toddler/Pages/default.aspx*

[**Just in Time Parenting: 12 Month Newsletter**](https://jitp.info/newsletter/month-12/)

*https://jitp.info/newsletters/month-12/*

[**Just in Time Parenting: 13-14 Month Newsletter**](https://jitp.info/newsletter/months-13-14/)

*https://jitp.info/newsletters/months-13-14/*

[**ASHA ProFind**](https://www.asha.org/profind/)

ASHA, the American Speech-Language-Hearing Association, is the professional organization for speech-language pathologists (specialist who helps with learning skills for feeding, speaking, and language) and audiologists (specialist who helps with hearing). This site can help you find a licensed speech, language, or hearing professional.

*https://www.asha.org/profind/*

[**Guide to seeking evaluation**](https://kidshealth.org/en/parents/special-ed-support.html)

This page from KidsHealth.org outlines the steps for finding testing for learning, mental health, or other needs for your child.

*https://kidshealth.org/en/parents/special-ed-support.html*

[**Illinois Early Learning Project**](https://illinoisearlylearning.org/)

The Illinois Early Learning Project is a source of research-based, reliable information on early care and education for families, caregivers, and teachers of young children in Illinois.

*https://illinoisearlylearning.org/*

**References**

1. Spitzer RL, Kroenke K, Williams JB, Löwe B. A brief measure for assessing generalized anxiety disorder: the GAD-7. Arch Intern Med. 2006;166(10):1092-7.

2. Kroenke K, Spitzer RL, Williams JB. The PHQ-9: validity of a brief depression severity measure. J Gen Intern Med. 2001;16(9):606-13.
