# Supplementary material for: The Founders’ 400 and Chicago Perinatal Origins of Disease study protocol: Following a prospective, longitudinal cohort from early pregnancy through two years of postnatal life
Source: PLoS One. 2025 Sep 29;20(9):e0332928. doi: 10.1371/journal.pone.0332928 (PMC12478913; doi:10.1371/journal.pone.0332928)
Supplement: S2 Appendix — (DOCX) [file pone.0332928.s002.docx]

**Appendix 2. Household Cleaning Practices Questionnaire**

1. What are the top 3-5 cleaning products do you use at home to clean your surfaces and bathrooms (names/brand of products;)?
   1. Could you upload pictures of the products labels?
2. What products do you specifically use to clean the surfaces in your baby’s room or your baby’s items?
   1. Could you upload pictures of the products labels?
3. What hand soap do you use for yourself and your baby?
   1. Could you upload pictures of the products labels?
4. What kind of cleaning wipes do you use?
   1. How often do you use these wipes? (Answers: daily, weekly, every two weeks, monthly, less often)
5. Do any of your cleaning products say “antimicrobial,” “antibacterial,” or “disinfectant”? use disinfectants to clean your home? Y/N
   1. If yes, which products?
   2. If yes, how often do you use these products? (Answers: daily, weekly, every two weeks, monthly, less often)
6. Do you have pets that live in the home?
   1. If yes, how many?
   2. If yes, what kind?
   3. If yes, how long have you had your pets?
7. What kind of building do you live in?
   1. Small apartment building
   2. Large apartment building/high rise
   3. Single family home
   4. Townhouse
   5. Other (describe)
8. What year was your house/building built?
9. Do you have a yard or outdoor space? Y/N
10. What size is your home (in square feet)?
11. How many adults live in your household?
12. How many children live in your household?
13. What kinds of flooring do you have in your home? Carpet, tile, hardwood, concrete, vinyl flooring, rugs, etc.
14. What kind of cooling/heating does your home have? Central air, radiator heat, window AC unit, wood stove, gas stove (for heat), Other, no heating or cooling systems
    1. How often do you use this appliance? (Answers: daily, weekly, every two weeks, monthly, less often)
15. Do you ever open your windows? Y/N
    1. If yes, how often? (Answers: daily, weekly, every two weeks, monthly, less often)
16. Do you have an air filtration system or air purifier?
    1. If yes, how often do you use it? (Answers: daily, weekly, every two weeks, monthly, less often)
17. Do you have a humidifier or de-humidifier?
    1. If yes, how often do you use it? (Answers: daily, weekly, every two weeks, monthly, less often)
18. What is the average temperature in your home currently?
19. What is the average humidity in your home currently?
20. Is there any visible or significant water damage in your home?
