# Supplementary material for: The Founders’ 400 and Chicago Perinatal Origins of Disease study protocol: Following a prospective, longitudinal cohort from early pregnancy through two years of postnatal life
Source: PLoS One. 2025 Sep 29;20(9):e0332928. doi: 10.1371/journal.pone.0332928 (PMC12478913; doi:10.1371/journal.pone.0332928)
Supplement: S3 Appendix — (DOCX) [file pone.0332928.s003.docx]

**Appendix 3. Trauma and Life Experiences Semi-Structured Interview**

Intergenerational childhood adversity, trauma, and life experiences are assessed using a semi-structured interview during pregnancy visit 3, postnatal visit 1 (6 weeks postpartum), and postnatal visit 4 (18 months postpartum). Interviews are conducted in-person or via phone or secure Zoom, audio-recorded, and transcribed. The trauma and life experiences interview guide, modified from the validated Psychosocial Assessment Tool, covers topics including abuse history, household disciplinary practices, and parental feelings toward their child, as outlined below [1-3].

Given the highly sensitive nature of topics explored in our semi-structured interviews, including experiences related to abuse, substance use, and mental health challenges, we have implemented several protocols to ensure participant safety and staff well-being. All interviewers complete a two-day training with a licensed clinical social worker with expertise in trauma-informed care who works closely with our team. These trainings center on active listening and strategies for navigating emotional distress in a supportive and non-judgmental manner, while also exploring the potential of challenging responses and guidance on when and how to escalate, if needed, for instance if a participant expresses that they or their child is in immediate danger. Trainings also include practical scenarios and live demonstration with our social worker to practice interview skills prior to working directly with participants. Research staff undergo follow-up training sessions with this licensed clinical social worker as needed every 3-6 months.

If significant psychosocial distress, mental health concerns, safety concerns, or unmet social needs are identified during an interview or any other study assessments, clinical providers are notified and participants are referred to appropriate resources, including a licensed social worker, mental health support, and/or other community resources (i.e. food pantries, domestic violence support programs, parenting education, peer support, employment services, etc.). We utilize Unite Us [4], a verified referral platform to provide closed-loop referrals in a secure method to reduce the risk of increased burden on participants to reach out to resources themselves, and to ensure a connection to the resource is confirmed. We also recognize the potential for secondary trauma among interviewers. Regular debriefing opportunities and access to mental health support services are provided to all research team members to mitigate potential emotional burden.

***Trauma and Life Experiences Semi-Structured Interview Guide***

**PRENATAL (Pregnancy Study Visit 3)**

*We will be asking you a series of questions about your social and familial history and current circumstances. Some of these questions are more sensitive. These questions are a part of this research study because a child’s environment is shaped so strongly by their caregivers; we want to better understand your stressors and support as a future caregiver. If any questions indicate that someone, such as an adult or child, could be in danger, we are mandated to follow local policies to ensure safety. However, we want this to feel like a safe space, and you can decline to answer if you feel uncomfortable at any time.*

Before we start, could you remind me who lives in your home with you? Is the father of the baby involved and/or do you have a romantic partner who is involved in the pregnancy?

1. How would you describe your childhood?
2. How safe did you feel safe as a child?
3. What made you feel unsafe as a child?
   1. What did discipline look like in your household when you were a child?
4. Would you (or do you) discipline your children in this way?
5. Who raised you as a child?
6. Do you know what child protective services is, or what our state calls DCFS? Was DCFS ever involved with you as a child?
   1. *Prompt:* Were you ever removed from living with your parents or primary caregiver(s)?
7. Has anyone currently living in your household been involved with “DCFS”?
8. Do you know what foster care is? Were you ever in foster care as a child?
9. Has anyone currently living in your household had interactions with the police or been arrested for any reason?
   1. *Prompt, if needed, to re-frame*: Have you or your partner ever called the police regarding an incident in your household?
   2. *Note to Research Assistant (RA): if hesitant to answer this question, can reframe as “can you tell me if anyone in your home has ever had any law enforcement contact for violent crimes or crimes against children?”*
10. Were any of your parents/guardians ever incarcerated?
11. Have you ever been incarcerated?

*(If this is via Zoom) I can't tell who may be around or in the home with you. It is your choice, but some of these questions are personal and people feel more comfortable answering them when they are alone. Are you alone? If not, are you able to answer these questions safely?*

1. Did you witness intimate partner violence as a child?
2. If they currently had a partner (answered on the medical/social hx questionnaire):
   1. How do you and your partner work out arguments?
   2. Do your arguments ever escalate into screaming, yelling, or name-calling?
      1. *Note to RA: pause between each action to allow time for response*
   3. Do your arguments ever escalate into hitting, kicking, slapping, pushing, or choking?
   4. Has your partner ever intimidated, threatened, or stalked you?
   5. Has there ever been a time where you felt unsafe in your relationship?
3. Have you or anyone in your current household ever used physical means to resolve conflict?
4. Have you ever experienced any kind of abuse?
   1. If yes, tell me more.
   2. *Prompts if needed:*
      1. Were you a victim of physical and/or sexual abuse as a child?
      2. As an adult?
      3. Was this reported to child protective services or law enforcement?
5. Does anyone who is currently living in your household have problems with drug or alcohol use?

*If yes (or asks for more description) then ask the below. Note to RA: pause between each item to allow time for response:*

- Is there anyone in your house who you worry drinks too much?
- Takes pills that aren’t prescribed to them or takes more pills than they should?
- Smokes marijuana/takes THC edibles every day or you feel relies too much on THC?
- Does other drugs (including those they may have to buy on the street)?

*These next few questions are about mental health:*

1. Does anyone currently living in your household have mental health issues, such as anger management or temper, depression, bipolar disorder, post-traumatic stress disorder, anxiety, or schizophrenia? *(Please read each item in list)*
   1. If yes, which diagnosis?
   2. If yes, does this person receive therapy or treatment for their mental health?
   3. Do they live in the home or will they be a part of your child’s life?
      1. If yes, will they be a caregiver for your child?
   4. If needed: do you feel like they are well-managed and getting the support they need?
2. *If other children:* have you experienced post-partum depression or psychosis after the birth of a child?
3. Have you ever thought about hurting or killing yourself?
   1. Have you ever thought about hurting or killing others?
   2. Have you ever engaged in any self-harm behaviors, such as cutting, scratching, hair pulling, etc.?
   3. If yes to any:
      1. Tell me more
      2. Are you feeling that way now or during this pregnancy?
         1. *For RA – if reported yes during pregnancy, report this to the obstetric provider*
   4. If yes but not anymore:
      1. If you were to feel that way again, who would you call or what would you do?
4. Could you describe any experiences where you felt you were treated differently than others?
   1. *Rephrase if needed:* Tell me about a time where someone treated you differently because of how you look.
   2. *Rephrase if needed:* Do you feel as if people treat you differently because of your identity (whether gender/sexual identity or racial/ethnic identity)?
5. Do you feel that you have positive social or family support?
   1. Who are your support systems?

*We have come to the end of the questions, is there anything that we’ve talked about today that you want to say more about or add to? Are there other resources or support you feel like you can benefit from?*

*If yes: We are happy to connect you with our resources here at Northwestern – I will have our social work team contact you.*

**POSTNATAL (Postnatal study visit 1)**

*We will be asking you a series of questions about your and your child’s history and current circumstances. Some of these questions are sensitive in nature. If any of our assessments indicate that you or a child is in imminent danger, we are required to follow local policies.*

1. What three words would you choose to describe your baby?
2. What is your favorite thing your baby does?
   1. What do you think causes your baby to do this?
   2. What do you do in response to this behavior?
3. What is your baby’s most frustrating behavior?
   1. Why do you think your baby behaves this way?
   2. What do you do in response to this behavior?
4. How does your baby communicate their needs/wants to you?
5. Who is the primary caretaker of the baby?
6. What are the childcare arrangements?
   1. *Prompts if needed*: does your baby go to daycare, do you have a babysitter, does family help care for your baby?
   2. Is this a formal or consistent arrangement?
7. What are the sleeping arrangements for your baby?
   1. *If needed* (i.e. if it’s not clear if they are bed sharing or room sharing):
      1. Do they sleep in their own bed or in bed with you?
      2. Is their bed/crib/bassinet in the same room as you or in another room?

*Again, these next few questions are more sensitive. These questions are a part of this research study because a child’s environment is shaped so strongly by their caregivers; we want to better understand your stressors and support as a future caregiver. These questions will be used for research purposes, and you can decline to answer if you feel uncomfortable at any time. Although this is for research, as mentioned earlier, if any questions indicate that someone, such as an adult or child, could be in danger, we are mandated to follow local policies to ensure safety.*

1. How do you respond when your baby misbehaves?
2. [If other children]: What does discipline look like in your home?
   1. *Prompt:* Do you or have you ever used physical discipline with your child? *(If additional prompt needed:* For example, have you ever spanked your baby? Do you or have you used objects (belts, paddles, etc.) on your baby?)
3. If anyone else watches baby: What does discipline look like from the other caregivers to your baby?
   1. If there is discipline, what kind? Do they spank your baby or use other objects to discipline your baby?
4. Have you ever noticed any bruises or injuries on your baby?
   1. If yes, please tell me more
5. Have you or any caregivers had difficulty affording concrete needs (housing, transportation, clothing, food/formula, etc.)?
   1. If yes, what are your sources of financial support (child support, unemployment, food pantry, LINK, housing choice voucher, family assistance, etc.)?
6. *If the child’s home has changed since interview #1 (or previous interview*): tell me more

about this.

1. Has DCFS been ever involved with your child/child(ren)?
   1. If yes, tell me more about this?
      1. *Prompts:* When did this happen, who was present, what happened afterwards?
   2. Was your child ever removed from your care or custody?
2. Do you have any thoughts about hurting or killing yourself?
   1. Do you have any thoughts about hurting or killing others?
   2. Are you engaging in any self-harm behaviors, such as cutting, scratching, hair pulling, etc.?
   3. If yes to any:
      1. Tell me more
      2. Are you feeling that way now or since the baby has been born?
   4. If yes but not anymore:
      1. If you were to feel that way again, who would you call or what would you do?
3. If they have a different partner than in interview #1 (or previous interview):

*(If same partner) I know these questions may have been asked of you before, but we generally ask these questions about your partner again.*

*(If this is via Zoom) I can't tell who may be around or in the home with you. It is your choice, but some of these questions are personal and people feel more comfortable answering them when they are alone. Are you alone? If not, are you able to answer these questions safely?*

- 1. How do and your partner work out arguments?
  2. Do your arguments ever escalate into screaming, yelling, or name-calling?
  3. Do your arguments ever escalate into hitting, kicking, slapping, pushing, or choking?
  4. Has your partner ever intimidated, threatened, or stalked you?
  5. Has there ever been a time when you felt unsafe in your relationship?

*If participant reports that she and partner have separated or divorced, please ask the appropriate prompts in the Interview Escalation Procedures document. You may need to ask the participant to remind you if the current partner is also the biological parent of the enrolled baby.*

**POSTNATAL (Postnatal study visit 4)**

*We will be asking you a series of questions about your and your child’s history and current circumstances. Some of these questions are sensitive in nature. If any of our assessments indicate that you or a child is in imminent danger, we are required to follow local policies.*

1. What three words would you choose to describe your toddler?
2. What is your favorite thing your toddler does?
   1. What do you think causes your toddler to do this?
   2. What do you do in response to this behavior?
3. What is your toddler’s most frustrating behavior?
   1. Why do you think your toddler behaves this way?
   2. What do you do in response to this behavior?
4. How does your toddler communicate their needs/wants to you?
5. Who is the primary caretaker of your child?
6. What are his/her childcare arrangements?
   1. *Prompts if needed:* does your toddler go to daycare, do you have a babysitter, does family help care for them?
   2. Is this a formal or consistent arrangement?

*Again, these next few questions are more sensitive. These questions are a part of this research study because a child’s environment is shaped so strongly by their caregivers; we want to better understand your stressors and support as a future caregiver. These questions will be used for research purposes, and you can decline to answer if you feel uncomfortable at any time. Although this is for research, as mentioned earlier, if any questions indicate that someone, such as an adult or child, could be in danger, we are mandated to follow local policies to ensure safety.*

1. What does discipline look like in your home?
2. When your toddler misbehaves (or has a non-preferred behavior of some sort?), how do you respond to them?
   1. *Prompt:* Do you or have you ever used physical discipline with your child?
   2. *If additional prompt needed:* For example, have you ever spanked your toddler? Do you or have you used objects (belts, paddles, etc.) on your child/children?
3. What does discipline look like from other caregivers?
   1. What does this look like? Do they spank your baby or use other objects to discipline your child?
4. Have you ever noticed any unexplained bruises or injuries on your child?
   1. If they say “yes, on their shins”, ask about any other locations
   2. If yes, please tell me more
5. Have you or any caregivers had difficulty affording concrete needs (housing, transportation, clothing, food/formula, etc.)?
   1. If yes, what are your sources of financial support (child support, unemployment, food pantry, LINK, housing choice voucher, family assistance, etc.)?
6. If the child’s home has changed since interview #1 (or previous interview): tell me more

about this.

1. Has DCFS been ever involved with your child/child(ren)?
   1. If yes, tell me more about this?
      1. *Prompts:* When did this happen, who was present, what happened afterwards?
   2. Was your child ever removed from your care or custody?
2. Do you have any thoughts about hurting or killing yourself?
   1. Do you have any thoughts about hurting or killing others?
   2. Are you engaging in any self-harm behaviors, such as cutting, scratching, hair pulling, etc.?
   3. If yes to any:
      1. Tell me more
      2. Are you feeling that way now or since the baby has been born?
   4. If yes but not anymore:
      1. If you were to feel that way again, who would you call or what would you do?
3. Partner Questions:

*If they have a different partner than in the previous interview, ask about the new partner or any changes that have occurred.*

*(If same partner)* *I know these questions may have been asked of you before, but we generally ask these questions about your partner again.*

*(If this is on Zoom) I can't tell who may be around or in the home with you. It is your choice, but some of these questions are personal and people feel more comfortable answering them when they are alone. Are you alone? If not, are you able to answer these questions safely?*

- 1. How do and your partner work out arguments?
  2. Do your arguments ever escalate into screaming, yelling, or name-calling?
  3. Do your arguments ever escalate into hitting, kicking, slapping, pushing, or choking?
  4. Has your partner ever intimidated, threatened, or stalked you?
  5. Has there ever been a time when you felt unsafe in your relationship?

*If participant reports that she and partner have separated or divorced, please ask the appropriate prompts in the Interview Escalation Procedures document. You may need to ask the participant to remind you if the current partner is also the biological parent of the enrolled baby.*

**References**

1. Pai AL, Patiño-Fernández AM, McSherry M, Beele D, Alderfer MA, Reilly AT, et al. The Psychosocial Assessment Tool (PAT2.0): psychometric properties of a screener for psychosocial distress in families of children newly diagnosed with cancer. J Pediatr Psychol. 2008;33(1):50-62.

2. Pierce MC, Kaczor K, Thompson R. Bringing back the social history. Pediatr Clin North Am. 2014;61(5):889-905.

3. Kazak AE, Barakat LP, Ditaranto S, Biros D, Hwang WT, Beele D, et al. Screening for psychosocial risk at pediatric cancer diagnosis: the psychosocial assessment tool. J Pediatr Hematol Oncol. 2011;33(4):289-94.

4. About Unite Us: Our Vision for Coordinated Community Health: Unite Us. 2025. Available from: uniteus.com/about-us.
