# Supplementary material for: The Founders’ 400 and Chicago Perinatal Origins of Disease study protocol: Following a prospective, longitudinal cohort from early pregnancy through two years of postnatal life
Source: PLoS One. 2025 Sep 29;20(9):e0332928. doi: 10.1371/journal.pone.0332928 (PMC12478913; doi:10.1371/journal.pone.0332928)
Supplement: S4 Appendix — (DOCX) [file pone.0332928.s004.docx]

**Appendix 4. Postnatal infant developmental assessments**

Standardized, validated measures assess early childhood neurodevelopment, language, communication, and behavior at five time points over the first two years of life (**Table 3**). Assessments are longitudinal and age-appropriate, with corrected postnatal age used as indicated. Most activities are performed remotely, except for in-person assessments at 12-15 months.

Much of the focus postnatally is on childhood neurodevelopment, assessed via the Ages and Stages Questionnaires, Third Edition (ASQ®-3) [1], Bayley Scales of Infant Development, Fourth Edition (Bayley™-4) [2], and Modified Checklist for Autism in Toddlers, Revised with Follow-up (M-CHAT-R/F) [3]. Beyond the ASQ®-3 and Bayley™-4, language and communication are assessed employing the Preschool Language Scales, Fifth Edition (PLS™-5) [4] screener and MacArthur-Bates Communicative Development Inventories (MCDI) Words and Sentences short form [5]. Lastly, early childhood behavioral regulation is assessed using the parent-reported Multidimensional Assessment Profiles Temper Loss Scale (MAPS-TL) [6-8] and the Early Regulation in Context Assessment with Family Culture Matters Activity (ERICA-FCM) [9] pragmatic observational assessment.

Video recording of the Bayley™-4 and ERICA-FCM in-person at the 12-month postnatal visit in-person (or recorded via Zoom, if performed remotely) permitted the study team to monitor the quality of these neurodevelopmental assessments, provide ongoing training and feedback to research staff, make iterative changes to protocol administration to ensure ease of administration and participant comfort across the longitudinal study period, and ensure reliability of scoring during periodic audits [9].

**References**

1. Singh A, Yeh CJ, Boone Blanchard S. Ages and Stages Questionnaire: a global screening scale. Bol Med Hosp Infant Mex. 2017;74(1):5-12.

2. Balasundaram P, Avulakunta ID. Bayley Scales Of Infant and Toddler Development. StatPearls. Treasure Island (FL): StatPearls Publishing, LLC.; 2023.

3. Robins DL, Casagrande K, Barton M, Chen CM, Dumont-Mathieu T, Fein D. Validation of the modified checklist for Autism in toddlers, revised with follow-up (M-CHAT-R/F). Pediatrics. 2014;133(1):37-45.

4. Hsiao YY, Qi CH, Hoy R, Dale PS, Stump GS, Davison MD, et al. Hierarchy and Reliability of the Preschool Language Scales-Fifth Edition: Mokken Scale Analysis. J Speech Lang Hear Res. 2021;64(10):3983-94.

5. Marchman VA, Dale PS. The MacArthur-Bates Communicative Development Inventories: updates from the CDI Advisory Board. Front Psychol. 2023;14:1170303.

6. Wiggins JL, Roy AK, Wakschlag LS. MAPping affective dimensions of behavior: Methodologic and pragmatic advancement of the Multidimensional Assessment Profiles scales. Int J Methods Psychiatr Res. 2023;32(S1):e1990.

7. Wakschlag LS, Briggs-Gowan MJ, Hill C, Danis B, Leventhal BL, Keenan K, et al. Observational Assessment of Preschool Disruptive Behavior, Part II: validity of the Disruptive Behavior Diagnostic Observation Schedule (DB-DOS). J Am Acad Child Adolesc Psychiatry. 2008;47(6):632-41.

8. Krogh-Jespersen S, Kaat AJ, Petitclerc A, Perlman SB, Briggs-Gowan MJ, Burns JL, et al. Calibrating temper loss severity in the transition to toddlerhood: Implications for developmental science. Applied Developmental Science. 2022;26(4):785-98.

9. Edwards RC, Planalp EM, Bosquet Enlow M, Akshoomoff N, Bodison SC, Brennan MB, et al. Capturing the complexity of child behavior and caregiver-child interactions in the HEALthy Brain and Child Development (HBCD) Study using a rigorous and equitable approach. Developmental Cognitive Neuroscience. 2024;69:101422.
