# Supplementary material for: The Founders’ 400 and Chicago Perinatal Origins of Disease study protocol: Following a prospective, longitudinal cohort from early pregnancy through two years of postnatal life
Source: PLoS One. 2025 Sep 29;20(9):e0332928. doi: 10.1371/journal.pone.0332928 (PMC12478913; doi:10.1371/journal.pone.0332928)
Supplement: S5 Appendix — (DOCX) [file pone.0332928.s005.docx]

**Appendix 5. Biologic and environmental specimen collection, processing, and storage**

*Urine*

Maternal urine samples are collected in early and late pregnancy during routine prenatal visits. Urine samples from infants are collected at 12-15 months of life during an in-person research visit (postnatal visit 3). Urine samples are aliquoted and frozen at -80^o^C.

*Blood*

Maternal blood samples are collected in early and late pregnancy, coordinated with routine prenatal lab draws, and at delivery. Cord blood is collected by delivery staff. If cord blood is unable to be collected immediately before placental delivery, a trained labor and delivery nurse will draw blood from any residual length of the umbilical cord or draw fetal blood from the placental vasculature within 30 minutes of delivery. Blood is drawn from infants once at 12-15 months of age by trained research phlebotomists or, if unavailable, with application of the Touch Activated Phlebotomy (TAP) II® Micro capillary blood self-collection device supervised by study personnel [1].

Blood is drawn in EDTA-coated tubes and stored at -4°C (or room temperature, for peripheral blood mononuclear cell [PBMC] isolation) until processing is performed. Blood is centrifuged, and plasma aliquots are separated and stored at -80ºC for future metabolomic analyses and inflammatory cytokine assays. PBMCs are isolated and stored in liquid nitrogen for future immunologic analysis. Whole blood is also collected in DNA/RNA shield blood collection tubes (Zymo Research) and stored at -80ºC for future microbiome and epigenetic analyses. Blood is processed within 90 minutes of collection, except for delivery specimens. Maternal plasma and cord blood collected at delivery is processed as soon as possible after collection, and typically within 12 hours of delivery, although processing up to 24 hours of delivery was considered acceptable.

*Placenta*

Placental tissue is stored at -4°C until sampling occurs typically within 24 hours of delivery, although sample collection up to 72 hours after delivery was considered acceptable. A trained pathology technician collects placental samples in 2cm^3^ segments from the inner 2/3 of the placental disc by radius in three separate areas. Each sample is further subdivided into three smaller specimens, generating nine samples per participant. Three of these samples are flash frozen in liquid nitrogen for future metabolomic analysis. Three additional placental samples are placed in RNA later solution and stored at -80ºC for future epigenetic analysis. The last three placental samples are used for formalin-fixed, paraffin-embedded blocks.[2] The blocks are sectioned and mounted on glass slides with thicknesses of 4-5µm and stained with hematoxylin and eosin for histologic analysis by a perinatal pathologist.

*Swabs*

Several types of swabs for microbiome and epigenetic analyses are collected from the mother, infant, and household members. Buccal swabs for epigenetic analysis are collected from the mother and infant at the delivery hospitalization and 12 months postpartum. Participating household members self-collect buccal swabs at 4-6 months postpartum at home. Buccal swabs are collected using the Sk-1 swabs with Simhelix Dri-Capsule system developed by Boca Scientific, Inc.

Oral (saliva), nasal, vaginal, and rectal swabs are collected for microbial analysis. The mother self-collects swabs orally, vaginally, and rectally at pregnancy visits 1 and 3 and postpartum visits 2 and 4. Research staff members obtain oral swabs and nasal swabs on the infant at the delivery hospitalization and instructs parents on how to collect these samples from their infants at postnatal visits 2 and 4. FLOQ® swabs (COPAN Diagnostics Inc.) are used for both oral and nasal collections; these swabs are routinely used for clinical purposes for respiratory virus testing via anterior nasal specimen collection per the protocol outlined by the Centers for Disease Control and Prevention [3]. Stool samples are collected from infants at these same timepoints. Other household members who consent to participate also self-collect (or with the help from a parent, in the case of child participation) oral (saliva), nasal, and rectal swabs. For microbiome specimens, participants place the swab tip or stool sample in a sterile tube containing DNA/RNA Shield solution (Zymo Research). Swabs and stool are frozen at -80^o^C upon receipt.

*Environmental specimens*

For participants who indicated they live with a pet(s) in their home, a stool sample is collected from all household pets at the second postnatal time point using the same procedure described above. Participants are instructed to provide the most recent stool sample possible, with all samples required to be less than 24 hours old. Pet stool will be stored for future analyses of the gut microbiome in early childhood.

Along with pet stool, tap water samples are also collected at the second postnatal time point. Participants collect water from their home in a sterile high density polyethylene bottle from the tap that they most use for cleaning dishes and drinking water. These samples are aliquoted and frozen at -80^o^C for long-term storage. Future analyses of tap water include measurement of perfluorochemicals, lead and other heavy metals, quaternary ammonium, and other chemicals from household cleaning products.

For longitudinal assessment of household dust, samples are collected once during pregnancy and twice postnatally. Participants collect samples at home, place them in a sterile sealed bag, and provide sample location information via survey (e.g., carpet vs. hardwood services, kitchen vs. bedrooms). Dust samples are weighed and aliquoted into 1.5mL cryovials in a sterile environment and stored at -80^o^C. Dust samples will be used to assess contamination from household cleaning products (e.g., quaternary ammonium compounds).

**References**

1. Collier AY, Molina RL. Maternal Mortality in the United States: Updates on Trends, Causes, and Solutions. Neoreviews. 2019;20(10):e561-e74.

2. Khong TY, Mooney EE, Ariel I, Balmus NC, Boyd TK, Brundler MA, et al. Sampling and Definitions of Placental Lesions: Amsterdam Placental Workshop Group Consensus Statement. Arch Pathol Lab Med. 2016;140(7):698-713.

3. CDC. Interim Guidelines for Collecting and Handling of Clinical Specimens for COVID-19 Testing. 2024. Available from: https://www.cdc.gov/covid/hcp/clinical-care/clinical-specimen-guidelines.html?CDC_AAref_Val=https://www.cdc.gov/coronavirus/2019-ncov/lab/guidelines-clinical-specimens.html.
