# Supplementary material for: The Founders’ 400 and Chicago Perinatal Origins of Disease study protocol: Following a prospective, longitudinal cohort from early pregnancy through two years of postnatal life
Source: PLoS One. 2025 Sep 29;20(9):e0332928. doi: 10.1371/journal.pone.0332928 (PMC12478913; doi:10.1371/journal.pone.0332928)
Supplement: S6 Appendix — (DOCX) [file pone.0332928.s006.docx]

**Appendix 6. Chart abstraction variables**

| **Prenatal** | Obstetric factors | Gravidity, parity, prior history of adverse pregnancy outcomes |
| --- | --- | --- |
|  | Gynecological factors | sexually transmitted infections, vaginal infections during the index pregnancy, etc. |
|  | Medical comorbidities | pre-gestational or gestational diabetes mellitus; cardiac, renal, or autoimmune disease; asthma or gastrointestinal disorders; hematologic, thrombotic, or rheumatologic conditions; depression, anxiety or other mental health condition, etc. |
|  | Vaccination history | COVID, influenza, DTaP, RSV vaccines |
|  | Prenatal record data | pre-pregnancy body mass index, gestational weight gain, prenatal labs |
|  | Medications | over-the-counter, prescription, or hospital administration |
|  | Antenatal care history | date and location of first prenatal visit, clinic name, utilization of antenatal fetal surveillance, and consultations with registered dietician, licensed social worker, mental health support services, or neonatologist antenatally |
| **Delivery**  **record** | Timing, route, and outcome of delivery | gestational age at birth, mode of delivery (vaginal, operative vaginal, cesarean birth), indication for delivery, livebirth with or without neonatal demise versus intrauterine fetal demise |
|  | Peridelivery interventions | group B streptococcus status, receipt of antibiotics intrapartum, receipt of antepartum betamethasone injection(s) or intravenous magnesium |
|  | Obstetric complications | placental abnormality (placenta previa, vasa previa, placenta accreta spectrum, placental abruption), acute fatty liver of pregnancy, gestational thrombocytopenia, peripartum hemorrhage, in addition to the prespecified obstetric outcomes |
| **Neonatal** | fetal sex, birthweight, birthweight percentile, Apgar scores, umbilical cord gas pH and base excess, delayed cord clamping, LATCH score on postpartum days 1-4 (if breast/chest feeding), and diagnosis of any congenital anomalies, in addition to the prespecified neonatal outcomes. | |
| **Pediatric** | anthropometric measures (height, weight), medical history including any physical or developmental conditions, medication history, surgical history, immunizations, allergies, hospitalizations, emergency room visits | |

*COVID—coronavirus disease 19; DTaP—diphtheria, tetanus, and pertussis; RSV—respiratory syncytial virus; LATCH—Latch on, audible swallowing, type/shape of nipple, comfort, hold positioning*
