# Supplementary material for: The Founders’ 400 and Chicago Perinatal Origins of Disease study protocol: Following a prospective, longitudinal cohort from early pregnancy through two years of postnatal life
Source: PLoS One. 2025 Sep 29;20(9):e0332928. doi: 10.1371/journal.pone.0332928 (PMC12478913; doi:10.1371/journal.pone.0332928)
Supplement: S7 Appendix — (DOCX) [file pone.0332928.s007.docx]

**Appendix 7. Analysis plan for key study aims/sub-aims**

| **AIM 1** | Characterize environmental, social, and biologic exposures and health status during pregnancy, infancy, and early childhood in a diverse cohort of mother-fetus/infant dyads. |
| --- | --- |
| **AIM 2** | Develop a longitudinal biorepository of clinical data, patient-reported data, biospecimens, and environmental samples for future study in a diverse cohort of mother-fetus/infant dyads. |

**Sub-aim 1a. Determine transgenerational epigenetic markers of maternal adversity and trauma and evaluate associations with early childhood development.**

| **Analysis 1** | ***Exposures*** | *As assessed using data coded from the Trauma and Life Experiences Semi-Structured Interview (pregnancy visit 3; see Appendix 3):*  Co-primary exposures   - Subtypes of childhood discipline (verbal only, physical only, mix of verbal and physical; question #3) - Ever experience any kind of abuse (yes/no, question #16)   Secondary exposures   - Witness intimate partner violence as a child (yes/no, question #16b) - Victim of physical and/or sexual abuse as a child (yes/no, question #16c) - Victim of physical and/or sexual abuse as an adult (yes/no, question #16c) |
| --- | --- | --- |
|  | ***Outcomes*** | Epigenetic profiles in:   - *the birthing parent*, in maternal whole blood and the buccal swab from pregnancy visit 1 - *the neonate*, in cord blood whole blood and the neonatal buccal swab collected during delivery admission - *the child*, in buccal swabs collected at postnatal visit 3   *Method of assessment:* Using previously validated methods [2], DNA from plasma and buccal swabs will be extracted and quantified with the Qubit Fluorometer (Thermo Fisher, Waltham, MA) and plated randomly across 96-well plates. The Northwestern University  Genomics (NUSeq) Core Facility will perform bisulfite modification using the EZ DNA Methylation Kit (Zymo Research, Irvine, CA), and measure DNAm with the Illumina Methylation EPIC Beadarray (Illumina, San Diego, CA) per the manufacturer’s protocol. Quality control (QC) and normalization of the resulting array data will be performed in R, and functional normalization will be utilized to remove technical variation [4]. |
|  | ***Analysis Plan*** | Analyses will be performed using similar methods of study collaborators with experience in evaluating epigenetic profiles associated with trauma exposure [2]. Robust linear regression will be performed associating the exposures and outcomes. We will estimate robust standard errors and produce partial residual plots in R based on exposure subgroups. For analyses of differential DNA methylation (DNAm) measurements, DNAm beta-values will be the dependent variable, exposure to abuse/violence will be the independent variable, and models will be adjusted for relevant covariates as outlined below. Buccal cell DNAm data and blood cell DNAm data will be analyzed separately, though we will also perform Pearson’s correlation to correlate methylation profiles in maternal plasma and buccal swabs, and cord blood plasma and neonatal buccal swabs. We will also use regression models to compare maternal buccal with neonatal and child buccal DNAm profiles, and maternal blood with cord blood DNAm profiles, to evaluate for transgenerational epigenetic alterations associated on maternal exposure to abuse. Multiple comparisons will be corrected for with Bonferroni-adjustment to the significance threshold. |
|  | ***Effect size calculation*** | Given the exploratory nature of this analysis, formal effect size calculations were not performed. In a similar study, our collaborators evaluated epigenetic in stress response genes in 26 children with abusive injuries (versus 52 with accidental injuries). Testing for buccal cell differential DNAm at 78 Cytosine-phosphate-Guanine (CpG) sites within the FKBP5 stress response gene locus, they identified 11 CpGs were differentially methylated (p<0.05) in buccal cells (n = 65 buccal swabs, 24 abused). Three of these remained statistically significant after Bonferroni-correction for 78 tests (p= 0.00064), and the children with abuse-related injuries had lower DNAm at all three of these CpGs compared to those with accidental injuries [2].  Estimating approximately 80% of our cohort will complete the Trauma and Life Experiences Semi-Structured Interview (n=350), less than 10% of our cohort would have to report experiencing any kind of abuse as a child for us to be able to detect significant differences in buccal cell DNAm. As approximately 25% of individuals report experiencing any kind of abuse in their childhood [11], we anticipate having adequate power to detect significant differences in buccal cell DNAm based on our cohort size. |
| **Analysis 2** | ***Exposures*** | *Epigenetic profiles* in individuals with and without the co-primary trauma history exposures (from Analysis 1), assessed in:   - *the birthing parent*, in maternal plasma and the buccal swab from pregnancy visit 1 - *the neonate*, in cord blood plasma and the neonatal buccal swab collected during delivery admission - *the child*, in buccal swabs collected at postnatal visit 3 |
|  | ***Outcomes*** | **Primary:** *Bayley Scales of Infant and Toddler Development, Fourth Edition (****Bayley****™****-4)*** *[*13], assessed at postnatal visit 3. Children with below average neurodevelopment, defined as a score <25^th^ percentile, will be compared to those with average neurodevelopment or greater, defined as a score ≥25^th^ percentile)  **Secondary, development**   - *Ages and Stages Questionnaires, Third Edition (ASQ®-3)* [14], assessed at postnatal visits 1, 2, 4 and 5. An abnormal, or high-risk, composite ASQ®-3 screen, will be defined as having a high-risk score on at least one of the five individual domains (communication, gross motor, fine motor, personal-social, and problem solving) on any ASQ®-3 questionnaire completed during the study, and compared to a low-risk screen, defined as the absence of a high-risk score on any ASQ®-3 domain during the study period. A high-risk score is defined as greater than or equal to two standard deviations below the mean; all other scores are considered low-risk. - *Modified Checklist for Autism in Toddlers, Revised with Follow-up (M-CHAT-R/F)* [15], assessed at postnatal visit 4. An abnormal, or high-risk, M-CHAT-R/F score will be defined as a score 8-20, and compared to a low-moderate risk score will be any score ≤7.   **Secondary, language and communication**   - *Preschool Language Scales, Fifth Edition (PLS™-5)* [16], assessed at postnatal visit 3. Children with a below average score, defined as greater than or equal to one standard deviation below the mean, will be compared to children with an average score or greater, defined as less than one standard deviation below the mean. - *MacArthur-Bates Communicative Development Inventories (MCDI) Words and Sentences* short form [17]. Children with below average language skills, defined as a score <25^th^ percentile, will be compared to those with average language skills or greater, defined as a score ≥25^th^ percentile)   **Secondary, behavior**   - *Multidimensional Assessment Profiles Temper Loss Scale (MAPS-TL)* [18, 19], assessed at postnatal visits 1 and 2 - *Early Regulation in Context Assessment with Family Culture Matters Activity (ERICA-FCM)* [20], assessed at postnatal visit 3 |
|  | ***Analysis Plan*** | Similar to Analysis 1 [2], robust linear regression will be performed associating the exposures and outcomes. We will estimate robust standard errors and produce partial residual plots in R based on presence/absence of each outcome. In a case-control analysis design, for analyses of differential DNA methylation (DNAm) measurements, DNAm beta-values will be the dependent variable, normal/abnormal development (for each neurodevelopmental assessment measure) will be the independent variable, and models will be adjusted for relevant covariates as outlined below. Buccal cell DNAm data and blood cell DNAm data will be analyzed separately. We will perform the analysis for all individuals in the cohort, then stratified by the presence or absence of maternal exposure to abuse/violence (from Analysis 1, Exposures). Multiple comparisons will be corrected for with Bonferroni-adjustment to the significance threshold. |
|  | ***Effect size calculation*** | Given the exploratory nature of this analysis, formal effect size calculations were not performed. However, as described in Sub-aim 1a Analysis 1, based on the sample sizes reported in other similar studies [21, 22], we anticipate having adequate power to detect significant differences in buccal cell DNAm based on our cohort size. |
| **Covariates** | In Analyses 1 and 2 for sub-aim 1b, we will consider the following covariates in adjusted models, depending on the specific exposure:   - **Demographics:** maternal age at enrollment, household income, education, insurance, tobacco and/or substance use during pregnancy - **Medical and obstetric history:** parity - **Gestational age** at maternal biospecimen collection (pregnancy visit 1), **child age** at buccal swab collection (postnatal visit 3) - **Fetal sex** as a biological variable, when appropriate. | |

**Sub-aim 1b. Evaluate the association of psychosocial stress, positive support structures, and social vulnerability with the metabolome in pregnancy and with maternal, neonatal, and early childhood outcomes.**

| **Analysis 1** | ***Exposures***  *(high vs. low)* | **S2 Table. Psychosocial stress, social support, and social vulnerability domains**   \|  \| **Domain** \| **Index or Survey Instrument** \| **High** \| **Low** \| \| --- \| --- \| --- \| --- \| --- \| \| **Social vulnerability** \| **Food insecurity** \| AHC-HRSN [1], *Food (…worried food will run out?)* \| Often or  sometimes true \| Never true \| \| SVAT, *Food access (…adequate access to healthy food?)* \| Not at all/somewhat \| Very much \| \| **Financial insecurity** \| AHC-HRSN, *Financial Strain* \| Very/somewhat hard to pay for basics \| Not hard to pay for basics \| \| AHC-HRSN, *Employment* \| Need help finding/keeping job \| Do not need job help \| \| SVAT, *Financial security (…have enough money to live comfortably?)* \| Not at all/somewhat \| Very much \| \| **Housing instability** \| AHC-HRSN, *Living Situation* \| Worried about/do not have steady housing \| Have steady housing \| \| SVAT, *Residence (…safe, stable place to sleep/store possessions?)* \| Not at all/somewhat \| Very much \| \| **Safety & exposure to violence** \| AHC-HRSN, *Safety (…anyone hurt you or threaten you with harm?)* \| Sometimes, often, or frequently \| Never or rarely \| \| SVAT, *Risk environments (exposed to…violence, drug use, criminal activity?)* \| Not at all/somewhat \| Very much \| \| **Psychosocial stress and social support** \| **Psychosocial Stress** \| Perceived Stress Scale [3], pregnancy visit 2 \| ≥14 \| <14 \| \| Prenatal Distress Questionnaire [5], pregnancy visit 3 \| Highest quartile \| Lower 3 quartiles \| \| Patient Health Questionnaire-9 [6], pregnancy visits 1 & 3 \| ≥10 \| <10 \| \| Generalized Anxiety Disorder-7 [7],  pregnancy visits 1 & 3 \| ≥10 \| <10 \| \| **Discrimination** \| Everyday Discrimination Scale (EDS) [8], pregnancy visit 2 \| Highest quartile \| Lower 3 quartiles \| \| **Social support** \| Multi-Dimensional Scale of Perceived Social Support [9, 10],  Pregnancy visit 2 \| Lowest  quartile \| Top 3 quartiles \| \| SVAT, *Social network (friends/family help when you need it?)* \| Not at all/somewhat \| Very much \| \| *AHC-HSRN—*Accountable Health Communities Health-Related Screening Tool [1], pregnancy visit 2; *SVAT—*Structural Vulnerability Assessment Tool [12], pregnancy visit 3 \| \| \| \| \| |
| --- | --- | --- | --- | --- | --- | --- | --- | --- | --- | --- | --- | --- | --- | --- | --- | --- | --- | --- | --- | --- | --- | --- | --- | --- | --- | --- | --- | --- | --- | --- | --- | --- | --- | --- | --- | --- | --- | --- | --- | --- | --- | --- | --- | --- | --- | --- | --- | --- | --- | --- | --- | --- | --- | --- | --- | --- | --- | --- | --- | --- | --- | --- | --- | --- | --- | --- | --- | --- | --- |
|  | ***Outcomes*** | **Plasma metabolome** at pregnancy visits 1 and 3, with specific attention to lipid, fatty acid, and branched chain amino acid metabolites  *Method of assessment:* Maternal plasma metabolite concentrations will be measured via targeted metabolomics using the MxP Quant 500 XL Biocrates assay kit,^136^ which in a single kit can quantify up to 1,019 metabolites from 39 biochemical classes with high accuracy and reproducibility.^136^ In hypothesis-driven pathway and network-based analyses, we will evaluate lipids, lipid-related metabolites, (polyunsaturated fatty acids, glycerophospholipids, sphingolipids, acylcarnitines, pro-inflammatory oxylipins) and small metabolites (branched-chain amino acids, other amino acid intermediates of central metabolism); these pathways are sufficiently broad but targeted to facilitate several analyses.^35-39^ Targeted assays will be performed on an Agilent and Sciex Triple Quadropole system at the West Coast Metabolomics Center.^119^ Methods for sample preparation and extraction, data acquisition, raw data processing, and metabolite annotation have been validated and described.^137^ We will balance sample batches by parity and exposure and outcome groups, and employ ComBat for batch correction.^138^ Lipids, acylcarnitines, and hexoses are measured over two injections by flow injection analysis-tandem mass spectrometry (FIA-MS/MS) in positive ionization modes. Amino acids, fatty acids, vitamins and cofactors, and other small metabolites are measured by liquid chromatography-tandem mass spectrometry (LC-MS/MS) from two injections in positive and negative ionization modes. The MxP Quant 500 XL Biocrates kit includes lyophilized human plasma quality control (QC) samples representing low, medium, and high concentration levels. Quality control samples will be injected as first, middle, and last samples of each LC-MS/MS run. The QC samples are used to assess the technical performance of the plate by reporting the accuracies of each metabolite at each of the three concentrations levels. Data from all injections are imported and quantified in Biocrates WebIDQ™ software. Analytes from LC-MS/MS and FIA-MS/MS are quantified using internal standards.^136^ Manual curation and data transformation will be performed.^113^ |
|  | ***Analysis Plan*** | First, we will stratify each exposure as a binary variable (high/low) and assess plasma metabolites as continuous measures (S2 Table). We will filter metabolomic data to remove low-variance molecular features and perform variance-stabilizing normalization across batches. We will estimate adjusted mean differences in metabolite concentrations in birthing people with in the high/low exposure subgroups for each exposure via *t* tests, then apply generalized linear models (GLM) to identify individual plasma metabolites at each time point with significantly different log-transformed relative abundances between NPS groups (high/low), accounting for clinical covariates and controlling for false discovery rate (FDR, Benjamini-Hochberg method, q<0.05). Then, heat map visualization will depict associations between metabolite concentrations and the exposures for normalized concentrations of the top 25 metabolites at pregnancy visits 1 and 3 associated with each exposure [23]. Interaction testing will assess whether the association of each exposure with plasma metabolite concentrations is modified by individual concentrations of inflammatory cytokines (*IL-1β, IL-4, IL-6, IL-8, IL-10, IFN-γ, TNF-α, IL-4, IL-10,* measured via the Meso Scale Discovery [24] platform), assessed as binary variables (high versus low, above/below the 50^th^ percentile). Where a significant interaction exists, we will perform stratified analyses for high/low inflammation.  In addition, we will explore:  1) longitudinal changes in plasma metabolome profiles (from pregnancy visits 1 to 3) associated with the social exposures using linear mixed-effects regression models [25]  2) pathway enrichment analysis mapping the metabolites for which social exposures explain at least 1% of the metabolite’s variance into known metabolic pathways using the KEGG database [23]  3) network analyses to simultaneously model metabolite correlations and social exposures phenotype associations [26] |
|  | ***Effect size calculation*** | Assuming 10% of participants do not provide a plasma sample and 10% will have missing survey data, we anticipate having 350 participants in the analytic sample, and assume 25% have high social vulnerability, high psychosocial stress, and low social support. We will have 90% power at a two-sided α=0.002 (roughly consistent with FDR adjustment for 25 metabolites for overall 5% Type 1 error) to detect unadjusted group mean differences of ~0.54 SD for each social exposure. |
| **Analysis 2** | ***Exposures*** | **Plasma metabolites** at pregnancy visits 1 and 3, with specific attention to lipid, fatty acid, and branched chain amino acid metabolites, and assessed using the same methods as described above in *Analysis Plan 1, Outcomes* |
|  | ***Outcomes***  *(present*  *vs. absent)* | **Obstetric**   - Composite adverse pregnancy outcome (APO) - Individual obstetric outcomes: hypertensive disorders of pregnancy, preterm birth, fetal growth restriction   **Neonatal**   - Composite neonatal morbidity - Small-for-gestational age - Large-for-gestational age - Additional neonatal outcomes (Table 5)   **Infancy and early childhood outcomes**   - Composite adverse childhood development - Additional early childhood outcomes (Table 5) |
|  | ***Analysis plan*** | We will evaluate plasma and placental metabolite concentrations as continuous variables, filtering metabolomic data to remove low-variance molecular features and perform variance-stabilizing normalization across batches. I will use GLM to assess the association between the relative abundance of plasma metabolites with that in the placenta. Then, we will apply log-binomial regression to identify the relative risk of having the presence versus absence of each obstetric, neonatal, and infancy/early childhood outcome (composite, individual subtypes), while accounting for clinical covariates, and controlling for FDR (Benjamini-Hochberg method, q<0.05), for each one-standard deviation (SD) increase in the log-transformed relative abundances of plasma metabolites.  For the association of plasma metabolites, assessed at each time point, with adverse obstetric, neonatal, and infancy/early childhood outcomes, we will perform tests of interaction by the social vulnerability, psychosocial stress, and social support exposures from sub-aim 1b *Analysis 1* and if significant, perform stratified analysis by each high/low social exposure (S2 Table). We will similarly perform heat map visualization to depict these associations; these heat maps will visually distinguish differences between which plasma metabolites are associated with placental histopathology, and how these are further differentiated with exposure to high/low social exposures. To evaluate groups (i.e. blocks) of correlated plasma metabolites and their association with adverse obstetric, neonatal, and infancy/early childhood outcomes (presence/absence of the composite outcome), we will test for the association of the respective metabolites with these outcomes using the HAIIA (Hierarchical All Against All) method [27] We will also conduct network analyses to complement individual metabolite analyses, to simultaneously model plasma metabolite correlations and the obstetric, neonatal, and infancy/early childhood outcomes [26].  Additionally, we will explore:  1) the *joint associations of the social exposures* in sub-aim 1b, Analysis 1 (social vulnerability, psychosocial stress, social support) and *plasma metabolites* with each composite outcome, to determine what plasma metabolites independently add, over and above the social factors alone, to the association with the obstetric, neonatal, and early childhood outcome  2) *latent profile analysis* to identify if profiles of social factors, inflammation, and/or plasma metabolites during pregnancy distinguish mother-child dyads with the composite outcomes. |
|  | ***Effect size calculation*** | Assuming 10% loss to follow-up among 440 enrolled participants, we estimate 400 participants will have paired plasma and outcome data. Assuming adjustment for 4 covariates, we will have 90% power at a two-sided α=0.002 (roughly consistent with FDR adjustment for 25 metabolites to maintain overall 5% Type 1 error) to detect a minimum relative risk of 1.44 for the composite obstetric outcome, 2.31 for the composite neonatal outcome, and 2.14 for the composite infancy/early childhood outcome per one-SD change in plasma metabolite concentrations. |
| **Covariates** | In Analyses 1 and 2 for sub-aim 1b, we will consider the following covariates in adjusted models, depending on the specific exposure:   - **Demographics:** maternal age, household income, education, insurance, tobacco and/or substance use during pregnancy - **Medical and obstetric history:** prior APO, parity, progesterone or aspirin use - **Gestational age** at biospecimen collection - **Fetal sex** as a biological variable, when appropriate. | |

**Sub-aim 1c. Identify placental markers of fetal growth restriction, and placental markers associated with early childhood growth and neurodevelopment.**

| **Analysis 1** | ***Exposures*** | **Cases of fetal growth restriction (FGR),** a*s defined in Table 5,* assessed via sonographic diagnoses identified from antenatal chart abstraction   - Any FGR (estimated fetal weight or abdominal circumference <10^th^ percentile per the Hadlock curve) - Severe FGR (estimated fetal weight <3^rd^ percentile per the Hadlock curve)   **Controls:** absence of FGR |
| --- | --- | --- |
|  | ***Outcomes*** | **Placental histopathology** *(see below)*   - *primary:* composite histopathologic outcome - *secondary:* individual histopathologic lesion subtypes  \| **Composite histopathologic outcome** \| **Presence of at least 1 of the following histopathologic lesions:** \| \| --- \| --- \| \| **Chronic placental inflammation** \| Chronic villitis, deciduitis, chorioamnionitis; Histiocytic intervillositis; Eosinophilic T-cell vasculitis \| \| **Acute placental inflammation** \| Maternal inflammatory response  Fetal inflammatory response \| \| **Maternal vascular perfusion** \| Accelerated villous maturation; Placental villous infarction; Decidual arteriopathy Retroplacental hemorrhage or hematoma  Persistent muscularization of basal plate arteriole  Atherosis, fibrinoid necrosis of maternal arterioles \| \| **Fetal vascular malperfusion** \| Avascular villi, intervillous thrombus, intramural fibrin deposition, delayed villous maturation, or hypervascular chorangiosis \| \| **Perivillous fibrin deposition** \| Focus of perivillous fibrin deposition  Massive perivillous fibrin deposition \|   **Placental metabolome**   - assessed via similar methods as described in Sub-aim 1b, Analysis 1, Outcomes) |
|  | ***Analysis Plan*** | ***Placental histopathology***  Pearson’s Chi-square or the Wilcoxon rank-sum test, as appropriate, will be used to compare baseline characteristics between cases and controls. The proportion of the presence or absence of the primary and secondary placental histopathologic outcomes will be compared in cases of FGR versus controls without FGR using Pearson’s Chi-square test. Using generalized estimating equation (GEE) regression models with a binomial link function, univariable and multivariable analyses will be performed to generate the covariate-unadjusted and adjusted odds and 95% confidence intervals (CI), respectively, of cases (versus controls) having the primary and secondary placental histopathologic outcomes. To address missing covariates, we will consider inverse probability weighting or multiple imputation. Univariate and multivariable analyses will be repeated in a pre-specified sensitivity analysis excluding individuals with pre-pregnancy BMI ≥30kg/m^2^, given the potential link between obesity and placental inflammation in prior literature [28, 29]. Due to the exploratory nature of this analysis, correction for multiple comparisons testing will not be performed.  **Placental epigenetics**  Analyses will be performed using similar methods as in the epigenetic analyses from Aim 1a [2]. Robust linear regression will be performed associating the exposures and outcomes. We will estimate robust standard errors and produce partial residual plots in R based on exposure subgroups. For analyses of differential DNA methylation (DNAm) measurements, placental DNAm beta-values will be the dependent variable, presence/absence of FGR will be the independent variable, and models will be adjusted for relevant covariates as outlined below. Multiple comparisons will be corrected for with Bonferroni-adjustment to the significance threshold.  ***Placental metabolome***  We will estimate adjusted mean differences in metabolite concentrations in cases versus controls via *t* tests, then apply generalized linear models (GLM) to identify individual placental metabolites with significantly different log-transformed relative abundances between cases of FGR and controls without FGR, accounting for clinical covariates and controlling for false discovery rate (FDR, Benjamini-Hochberg method, q<0.05). Then, heat map visualization will depict associations between metabolite concentrations and the exposures for normalized concentrations of the top 25 metabolites associated with the presence versus absence of FGR [23]. |
|  | ***Effect size calculation*** | Assuming 10% of participants are lost to follow-up at delivery and 10% of placentas are unable to be sampled at delivery, we anticipate analyzing 350 placentas. Of those, 10% will be cases of FGR (35 placentas).  **Placental histopathology**  Assuming the composite histopathologic outcome occurs in 60% of FGR cases [30, 31], we will have 90% power at a two-sided alpha of 0.05 to detect 52% greater odds of the presence (versus absence of) the composite histopathologic outcome in cases of FGR.  **Placental metabolomics**  Assuming 35 placentas with FGR and adjustment for 4 covariates, we will have 90% power at a two-sided α=0.002 (roughly consistent with FDR adjustment for 25 metabolites to maintain overall 5% Type 1 error) to detect an R^2^ of 0.0.49 (i.e., large effect size, f2, 0.98) for associations between placental metabolites with FGR. Based on this same sample size, power, and two-sided α, I will be able to detect a minimum 78% higher relative risk of FGR per one-SD change in placental metabolite concentrations (Table 6). |
| **Analysis 2** | ***Exposures*** | - Placental histopathologic lesion subtypes identified in Analysis 1 to be associated with FGR (any, severe) - Placental epigenetic profiles identified in Analysis 1 to be associated with FGR (any, severe) - Placental metabolomic profiles identified in Analysis 1 to be associated with FGR (any, severe) |
|  | ***Outcomes*** | **Primary:** *Bayley Scales of Infant and Toddler Development, Fourth Edition (****Bayley****™****-4****)* [13], assessed at postnatal visit 3. Children with below average neurodevelopment, defined as a score <25^th^ percentile, will be compared to those with average neurodevelopment or greater, defined as a score ≥25^th^ percentile)  **Secondary, development**   - *Ages and Stages Questionnaires, Third Edition (ASQ®-3)* [14], assessed at postnatal visits 1, 2, 4 and 5. An abnormal, or high-risk, composite ASQ®-3 screen, will be defined as having a high-risk score on at least one of the five individual domains (communication, gross motor, fine motor, personal-social, and problem solving) on any ASQ®-3 questionnaire completed during the study, and compared to a low-risk screen, defined as the absence of a high-risk score on any ASQ®-3 domain during the study period. A high-risk score is defined as greater than or equal to two standard deviations below the mean; all other scores are considered low-risk. - *Modified Checklist for Autism in Toddlers, Revised with Follow-up (M-CHAT-R/F)* [15], assessed at postnatal visit 4. An abnormal, or high-risk, M-CHAT-R/F score will be defined as a score 8-20, and compared to a low-moderate risk score will be any score ≤7.   **Secondary, language and communication**   - *Preschool Language Scales, Fifth Edition (PLS™-5)* [16], assessed at postnatal visit 3. Children with a below average score, defined as greater than or equal to one standard deviation below the mean, will be compared to children with an average score or greater, defined as less than one standard deviation below the mean. - *MacArthur-Bates Communicative Development Inventories (MCDI) Words and Sentences* short form [17]. Children with below average language skills, defined as a score <25^th^ percentile, will be compared to those with average language skills or greater, defined as a score ≥25^th^ percentile) |
|  | ***Analysis Plan*** | **Placental histopathology**  Pearson’s Chi-square or the Wilcoxon rank-sum test, as appropriate, will be used to compare baseline characteristics between individuals with and without the composite placental histopathologic outcome. For each placental histopathologic exposure (composite, individual subtypes), the proportion of children with average or greater/low-risk scores for the primary and secondary assessments/outcomes will be assessed using Pearson’s Chi-square test. Using generalized estimating equation (GEE) regression models with a binomial link function, univariable and multivariable analyses will be performed to generate the covariate-unadjusted and adjusted odds and 95% confidence intervals (CI), respectively, of individuals with (versus without) the composite and individual placental histopathologic outcomes having a child with below average/high-risk score(s) for the primary and secondary neurodevelopmental assessments/outcomes. To address missing covariates, we will consider inverse probability weighting or multiple imputation. We will then repeat the univariate and multivariable analyses using data from mother-child dyads in which a sonographic FGR diagnosis was not made antenatally, to determine if different associations exist between placental histopathology with abnormal early childhood neurodevelopment in children who were, and were not, growth restricted in utero.  We will conduct a sensitivity analysis excluding individuals with pre-pregnancy BMI ≥30kg/m^2^, given the potential link between obesity and placental inflammation in prior literature [28, 29]. Based on expert recommendation and clinical differences observed in early childhood development by child sex, we will examine models stratified by child sex (male versus female). All statistical analyses will be conducted using R. Due to the exploratory nature of this analysis, correction for multiple comparisons testing will not be performed.  **Placental metabolome**  We will evaluate placental metabolite concentrations as continuous variables, filtering metabolomic data to remove low-variance molecular features and perform variance-stabilizing normalization across batches. Then, we will apply log-binomial regression in univariate and multivariate analysis to identify the relative risk of having a child with below average/high-risk score(s) for the primary and secondary neurodevelopmental assessments/outcomes, while accounting for clinical covariates, for each one-standard deviation (SD) increase in the log-transformed relative abundances of placental metabolites. We will control for FDR using the Benjamini-Hochberg method (q<0.05).  In an exploratory analysis, recognizing sample size limitations, we will repeat the univariate and multivariable analyses using data from mother-child dyads in which a sonographic FGR diagnosis was not made antenatally, to determine if different associations exist between placental metabolite profiles with abnormal early childhood neurodevelopment in children who were, and were not, growth restricted in utero. We will further stratify models by child sex (male versus female). |
|  | ***Effect size calculation*** | Assuming 10% of participants are lost to follow-up at delivery and 10% of placentas are unable to be sampled at delivery, we anticipate analyzing 350 placentas. Of children with corresponding placental data and completed the **Bayley**™**-4** in our cohort, we estimate 10% of the children in our cohort (35 children) will have below average neurodevelopment (i.e. score below the 25^th^ percentile on the **Bayley**™**-4**.)  **Placental histopathology**  Assuming the composite histopathologic outcome occurs in 39% of pregnancies [32, 33], we will have 90% power at a two-sided alpha of 0.05 to detect 50% greater odds of having the presence (versus absence of) the composite histopathologic outcome in children with below average neurodevelopment.  **Placental metabolomics**  Assuming 35 placentas with FGR and adjustment for 4 covariates, we will have 90% power at a two-sided α=0.002 (roughly consistent with FDR adjustment for 25 metabolites to maintain overall 5% Type 1 error) to detect an R^2^ of 0.0.49 (i.e., large effect size, f2, 0.98) for associations between placental metabolites with FGR. Based on this same sample size, power, and two-sided α, I will be able to detect a minimum 78% higher relative risk of a child having below average neurodevelopment per one-SD change in placental metabolite concentrations (Table 6). |
| **Covariates** | In Analyses 1 and 2 for sub-aim 1b, we will consider the following covariates in adjusted models, depending on the specific exposure:   - **Demographics:** maternal age, household income, education, insurance, tobacco and/or substance use during pregnancy, maternal depression or anxiety (at any point pre-pregnancy, during pregnancy, or through 8 weeks postpartum by medical records or self-report) - **Medical and obstetric history:** prior APO, parity, progesterone or aspirin use, antenatal corticosteroid exposure, delivery indication (spontaneous or medically indicated), group B streptococcus status), neonatal birthweight - **Gestational age** at biospecimen collection - **Fetal sex** as a biological variable, when appropriate   *Note:* Although we will report the distributions of hypertensive disorders (chronic hypertension, gestational hypertension, or preeclampsia), pre-pregnancy body mass index (BMI, both as a continuous and categorical measure), and pregestational or gestational diabetes in the analytic cohort, these maternal factors were not included as covariates as they may contribute to placental histopathology [28, 34-38]. | |

**Sub-aim 1d. Determine the impact of microbiota composition of household members and pets on the development of the gut microbiome of young children.**

| Analysis | ***Exposures*** | Gut microbiome composition in:   - household members, assessed using rectal swabs (or stool sample) at postnatal visit 2 - Gut microbiome composition in pets, assessed in stool samples collected at postnatal visit 2 |
| --- | --- | --- |
|  | ***Outcomes*** | Gut microbiome composition in children, assessed in stool samples collected at postnatal visit 2 and 4  *Methods of microbiome assessment:* We will perform DNA sequencing and analysis according to the protocols used by the Human Microbiome Project [39-41]. We will isolate DNA using the MoBio PowerSoil DNA Isolation Kit (MO BIO Laboratories, Carlsbad CA) following the manufacturer’s protocol, then store extracted DNA samples in solution at − 80 °C until sequencing. Using the Roche 454 GS FLX, pyrosequencing of V1V3 and V3V5 variable regions of the 16S rRNA gene will be performed. For sequences that pass quality filters, they will be classified using the Ribosomal Database Project naïve bayesian classifier, version 2.5 with training set 10 [42]. For the V1V3 and V3V5 variable regions, each sample will be subsampled to the lowest number of read counts among samples in the data set. |
|  | ***Analysis Plan*** | We will compare Shannon diversity in stool samples (or rectal swabs, as appropriate) between paired household member-child dyads, and separately pet-child dyads, using the Wilcoxon signed rank test. We will then calculate and compare beta diversity (Bray–Curtis dissimilarity), which will be visualized using non-metric multi-dimensional scaling (NMDS) plots in R [43, 44]. We will generate Pearson correlation coefficients for the taxa detected in the pair of household member (or pet) and child stool samples/swabs obtained at visit 2, and separately for child stool samples/swabs obtained at visit 4. Stacked bar charts will be generated to illustrate the taxonomic composition of the samples [45]. After removing taxa with fewer than five reads detected, we will perform linear discriminant analysis effect size (LEfSe) using the remaining data to determine whether there were taxa that were preferentially detected in the household member (versus child), or pet (versus child) [46]. |
| **Covariates** | We will consider the following covariates in adjusted models, depending on the specific exposure:   - **Demographics:** household income, maternal education, insurance, tobacco and/or substance use by the enrolled parent - **Child age** at biospecimen/environmental specimen collection - **Child sex** as a biological variable | |

**Sub-aim 1e. Evaluate the association of household dust and environmental chemicals (e.g., lead, quaternary ammounium compounds), in the context of housing and cleaning practices, with i) detection in human biospecimens, ii) infant microbiota/resistome, and iii) early childhood wheezing.**

| **Analysis 1** | ***Exposures*** | *In household dust,* assessed at pregnancy visit 2:   - quaternary ammonium compounds   *Method of assessment:* high-performance liquid chromatography-mass spectrometry |
| --- | --- | --- |
|  | ***Outcomes*** | *Maternal plasma concentrations* (continuous variable), assessed at pregnancy visit 3, of:   - quaternary ammonium compounds   *Cord blood plasma concentrations* (continuous variable), obtained at birth, of:   - quaternary ammonium compounds   *Method of assessment:* high-performance liquid chromatography-mass spectrometry |
|  | ***Analysis Plan*** | We will perform Pearson correlation to measure the strength of the relationship between concentrations of quaternary ammonium compounds in household dust with that in maternal plasma, and separately in cord blood plasma. We will also perform Pearson correlation to determine the strength of the correlation between maternal plasma and cord blood plasma concentrations of quaternary ammonium compounds. This analysis will be performed first for the overall cohort, then stratified by subgroups based on reported use (versus no use) of at least one household cleaning product that contains quaternary ammonium compounds. |
| **Analysis 2** | ***Exposures*** | *In household tap water* (continuous variable), assessed at postnatal visit 2   - lead - quaternary ammonium compounds   *In household dust,* assessed at pregnancy visit 2 and postnatal visits 2 and 4:   - quaternary ammonium compounds   *Method of assessment:* high-performance liquid chromatography-mass spectrometry |
|  | ***Outcomes*** | Gut microbiome in children, assessed in infant stool samples collected at delivery admission and postnatal visits 2 and 4.  *Method of microbiome assessment:* as described in Aim 1d |
|  | ***Analysis Plan*** | We will generate Pearson correlation coefficients for:   - concentrations of the three chemical exposures assessed in household tap water at postnatal visit 2 with the taxa detected in the child stool sample obtained at postnatal visit 2 - concentrations of quaternary ammonium compounds in household dust at pregnancy visit 2 with the taxa detected in the neonatal stool sample obtained at the delivery admission - concentrations of quaternary ammonium compounds in household dust at pregnancy visit 2 with the taxa detected in the child stool sample at postnatal visit 2 - concentrations of quaternary ammonium compounds in household dust at pregnancy visit 4 with the taxa detected in the child stool sample at postnatal visit 4   Stacked bar charts will be generated to illustrate the taxonomic composition of the samples [45]. After removing taxa with fewer than five reads detected, we will perform linear discriminant analysis effect size (LEfSe) using the remaining data to determine whether there were taxa that were preferentially detected in the household tap water, dust, and/or child stool samples [46].  To assess changes in the child’s gut microbiome overtime based on exposure to quaternary ammonium compounds, or lack of exposure to quaternary, we will calculate and compare beta diversity (Bray–Curtis dissimilarity), which will be visualized using non-metric multi-dimensional scaling (NMDS) plots in R [43, 44], at the delivery admission, postnatal visit 2, and postnatal visit 4 time points. We will further compare the beta-diversity in the subgroup of households with reported use (versus no use) of at least one household cleaning product that contains quaternary ammonium compounds. |
| **Analysis 3** | ***Exposures*** | *In household tap water* (continuous variable), assessed at postnatal visit 2   - lead - quaternary ammonium compounds   *In household dust,* assessed at pregnancy visit 2 and postnatal visits 2 and 4   - quaternary ammonium compounds   *Method of assessment:* high-performance liquid chromatography-mass spectrometry |
|  | ***Outcomes*** | Early childhood wheezing (present, ≥1 episode before age 2 versus absent, 0 episodes), assessed at postnatal visit 5, as reported by the birthing parent on the medical and medication questionnaire administered on behalf of the enrolled child and other children in the household |
|  | ***Analysis Plan*** | We will report descriptive statistics of the three chemical exposures assessed in household tap water, and concentrations of quaternary ammonium compounds in household dust. We will employ generalized linear models to evaluate the association of early childhood wheezing at each timepoint (present versus absent) with concentrations of the chemical exposures in household tap water and/or dust. |
|  | ***Effect size calculation*** | Of 440 mother-child dyads recruited, we assume 10% loss to follow up of children up to age 2 years, and 80% (n=350) of study participants will return household water and dust samples.  We further estimate 70% of children will have exposure to lead-contaminated drinking water (n=280), and 19% of exposed children use unfiltered tap water as their primary drinking source [47] and thus will have elevated blood lead levels.  In a pilot study of 92 individuals in our cohort, 17 (18%) reported use of at least one household cleaning product that definitively contains quaternary ammonium compounds (Tillema et al., abstract presented at the Indoor Air 2024: 18th International Conference of the International Society of Indoor Air Quality and Climate. July 7-11, 2024; Honolulu, HI.)  Estimating 25% of children [48, 49] in our cohort will have at least 1 episode of early childhood wheezing before age 2, we will have 90% power at a two-sided alpha of 0.05 to detect a:   - 24% greater odds of early childhood wheezing associated with exposure to high lead concentrations in household tap water - 25% odds of early childhood wheezing associated with exposure to quaternary ammonium compounds in household tap water or dust |
| **Covariates** | In Analyses 1-3 for sub-aim 1e, we will consider the following covariates in adjusted models, depending on the specific exposure:   - **Demographics:** household income, maternal education, insurance, tobacco and/or substance use by the enrolled parent - **Child age** at environmental specimen collection - **Child sex** as a biological variable | |

**References**

1. Billioux A, Verlander K, Anthony S, Alley D. Standardized screening for health-related social needs in clinical settings: The accountable health communities screening tool. National Academy of Medicine. Washington, DC. 2017.

2. Everson TM, Kaczor K, Makoroff K, Meyers G, Rosado N, Charleston E, et al. Epigenetic differences in stress response gene FKBP5 among children with abusive vs accidental injuries. Pediatric Research. 2023;94(1):193-9.

3. Cohen S, Kamarck T, Mermelstein R. A global measure of perceived stress. J Health Soc Behav. 1983;24(4):385-96.

4. Fortin J-P, Labbe A, Lemire M, Zanke BW, Hudson TJ, Fertig EJ, et al. Functional normalization of 450k methylation array data improves replication in large cancer studies. Genome Biology. 2014;15(11):503.

5. Ibrahim SM, Lobel M. Conceptualization, measurement, and effects of pregnancy-specific stress: review of research using the original and revised Prenatal Distress Questionnaire. J Behav Med. 2020;43(1):16-33.

6. Kroenke K, Spitzer RL, Williams JB. The PHQ-9: validity of a brief depression severity measure. J Gen Intern Med. 2001;16(9):606-13.

7. Spitzer RL, Kroenke K, Williams JB, Löwe B. A brief measure for assessing generalized anxiety disorder: the GAD-7. Arch Intern Med. 2006;166(10):1092-7.

8. Williams DR, Yan Y, Jackson JS, Anderson NB. Racial Differences in Physical and Mental Health: Socio-economic Status, Stress and Discrimination. J Health Psychol. 1997;2(3):335-51.

9. Zimet GD, Powell SS, Farley GK, Werkman S, Berkoff KA. Psychometric characteristics of the Multidimensional Scale of Perceived Social Support. J Pers Assess. 1990;55(3-4):610-7.

10. Kim H, Wildeman C, Jonson-Reid M, Drake B. Lifetime Prevalence of Investigating Child Maltreatment Among US Children. Am J Public Health. 2017;107(2):274-80.

11. Brown CL, Yilanli M, Rabbitt AL. Child Physical Abuse and Neglect. StatPearls. Treasure Island (FL): StatPearls Publishing, LLC.; 2025.

12. Bourgois P, Holmes SM, Sue K, Quesada J. Structural Vulnerability: Operationalizing the Concept to Address Health Disparities in Clinical Care. Acad Med. 2017;92(3):299-307.

13. Balasundaram P, Avulakunta ID. Bayley Scales Of Infant and Toddler Development. StatPearls. Treasure Island (FL): StatPearls Publishing, LLC.; 2023.

14. Singh A, Yeh CJ, Boone Blanchard S. Ages and Stages Questionnaire: a global screening scale. Bol Med Hosp Infant Mex. 2017;74(1):5-12.

15. Robins DL, Casagrande K, Barton M, Chen CM, Dumont-Mathieu T, Fein D. Validation of the modified checklist for Autism in toddlers, revised with follow-up (M-CHAT-R/F). Pediatrics. 2014;133(1):37-45.

16. Hsiao YY, Qi CH, Hoy R, Dale PS, Stump GS, Davison MD, et al. Hierarchy and Reliability of the Preschool Language Scales-Fifth Edition: Mokken Scale Analysis. J Speech Lang Hear Res. 2021;64(10):3983-94.

17. Marchman VA, Dale PS. The MacArthur-Bates Communicative Development Inventories: updates from the CDI Advisory Board. Front Psychol. 2023;14:1170303.

18. Wiggins JL, Roy AK, Wakschlag LS. MAPping affective dimensions of behavior: Methodologic and pragmatic advancement of the Multidimensional Assessment Profiles scales. Int J Methods Psychiatr Res. 2023;32(S1):e1990.

19. Wakschlag LS, Briggs-Gowan MJ, Hill C, Danis B, Leventhal BL, Keenan K, et al. Observational Assessment of Preschool Disruptive Behavior, Part II: validity of the Disruptive Behavior Diagnostic Observation Schedule (DB-DOS). J Am Acad Child Adolesc Psychiatry. 2008;47(6):632-41.

20. Edwards RC, Planalp EM, Bosquet Enlow M, Akshoomoff N, Bodison SC, Brennan MB, et al. Capturing the complexity of child behavior and caregiver-child interactions in the HEALthy Brain and Child Development (HBCD) Study using a rigorous and equitable approach. Developmental Cognitive Neuroscience. 2024;69:101422.

21. Gomaa N, Konwar C, Gladish N, Au-Young SH, Guo T, Sheng M, et al. Association of Pediatric Buccal Epigenetic Age Acceleration With Adverse Neonatal Brain Growth and Neurodevelopmental Outcomes Among Children Born Very Preterm With a Neonatal Infection. JAMA Netw Open. 2022;5(11):e2239796.

22. Stoccoro A, Conti E, Scaffei E, Calderoni S, Coppedè F, Migliore L, et al. DNA Methylation Biomarkers for Young Children with Idiopathic Autism Spectrum Disorder: A Systematic Review. International Journal of Molecular Sciences. 2023;24(11):9138.

23. Beuchel C, Becker S, Dittrich J, Kirsten H, Toenjes A, Stumvoll M, et al. Clinical and lifestyle related factors influencing whole blood metabolite levels - A comparative analysis of three large cohorts. Mol Metab. 2019;29:76-85.

24. Meso Scale Diagnostics, LLC. Meso Scale Discovery. 2025. Available from: https://www.mesoscale.com/en.

25. Chong J, Xia J. MetaboAnalystR: an R package for flexible and reproducible analysis of metabolomics data. Bioinformatics. 2018;34(24):4313-4.

26. Scholtens DM, Bain JR, Reisetter AC, Muehlbauer MJ, Nodzenski M, Stevens RD, et al. Metabolic Networks and Metabolites Underlie Associations Between Maternal Glucose During Pregnancy and Newborn Size at Birth. Diabetes. 2016;65(7):2039-50.

27. Ghazi AR, Sucipto K, Rahnavard A, Franzosa EA, McIver LJ, Lloyd-Price J, et al. High-sensitivity pattern discovery in large, paired multiomic datasets. Bioinformatics. 2022;38(Suppl 1):i378-i85.

28. Layden AJ, Bertolet M, Parks WT, Adibi JJ, Roberts JM, Catov JM. Prepregnancy obesity and risk of placental inflammation at term: a selection bias analysis. Ann Epidemiol. 2023;86:25-33.e7.

29. Howell KR, Powell TL. Effects of maternal obesity on placental function and fetal development. Reproduction. 2017;153(3):R97-r108.

30. Shmueli A, Mor L, Blickstein O, Sela R, Weiner E, Gonen N, et al. Placental pathology in pregnancies with late fetal growth restriction and abnormal cerebroplacental ratio. Placenta. 2023;138:83-7.

31. Spinillo A, Gardella B, Adamo L, Muscettola G, Fiandrino G, Cesari S. Pathologic placental lesions in early and late fetal growth restriction. Acta Obstet Gynecol Scand. 2019;98(12):1585-94.

32. Romero R, Kim YM, Pacora P, Kim CJ, Benshalom-Tirosh N, Jaiman S, et al. The frequency and type of placental histologic lesions in term pregnancies with normal outcome. Journal of Perinatal Medicine. 2018;46(6):613-30.

33. Goldstein JA, Gernand AD, Gallagher K, Shanes ED, Bebell LM, Yee LM. Defining Appropriate Comparator Populations for Placental Pathology for Pregnant People With HIV. Int J Surg Pathol. 2024:10668969241295351.

34. Pantham P, Aye IL, Powell TL. Inflammation in maternal obesity and gestational diabetes mellitus. Placenta. 2015;36(7):709-15.

35. Louwen F, Kreis N-N, Ritter A, Yuan J. Maternal obesity and placental function: impaired maternal–fetal axis. Archives of Gynecology and Obstetrics. 2024;309(6):2279-88.

36. Kovo M, Bar J, Schreiber L, Shargorodsky M. The relationship between hypertensive disorders in pregnancy and placental maternal and fetal vascular circulation. Journal of the American Society of Hypertension. 2017;11(11):724-9.

37. Bhojwani K, Agrawal A. Study of Histopathological Changes in the Placenta in Preeclampsia. Cureus. 2022;14(10):e30347.

38. Huynh J, Dawson D, Roberts D, Bentley-Lewis R. A systematic review of placental pathology in maternal diabetes mellitus. Placenta. 2015;36(2):101-14.

39. Evaluation of 16S rDNA-based community profiling for human microbiome research. PLoS One. 2012;7(6):e39315.

40. Aagaard K, Petrosino J, Keitel W, Watson M, Katancik J, Garcia N, et al. The Human Microbiome Project strategy for comprehensive sampling of the human microbiome and why it matters. Faseb j. 2013;27(3):1012-22.

41. Huttenhower C, Gevers D, Knight R, Abubucker S, Badger JH, Chinwalla AT, et al. Structure, function and diversity of the healthy human microbiome. Nature. 2012;486(7402):207-14.

42. Wang Q, Garrity GM, Tiedje JM, Cole JR. Naive Bayesian classifier for rapid assignment of rRNA sequences into the new bacterial taxonomy. Appl Environ Microbiol. 2007;73(16):5261-7.

43. Roberts D. labdsv: ordination and multivariate analysis for ecology.: CRAN-The Comprehensive R Archive Network; 2023 [R package version 2.1-0.:[

44. Oksanen J, Simpson G, Blanchet F, Kindt R, Legendre P, Minchin P, et al. Vegan: community ecology package. 2025. Available from: https://cran.r-project.org/web/packages/vegan/index.html.

45. Sievert C, Parmer C, Hocking T, Chamberlain S, Ram K, Corvellec M, et al. Plotly: create interactive web graphics via ‘plotly.js’: CRAN-The Comprehensive R Archive Network. 2024. Available from: https://cran.r-project.org/web/packages/plotly/index.html.

46. Segata N, Izard J, Waldron L, Gevers D, Miropolsky L, Garrett WS, et al. Metagenomic biomarker discovery and explanation. Genome Biol. 2011;12(6):R60.

47. Huynh BQ, Chin ET, Kiang MV. Estimated Childhood Lead Exposure From Drinking Water in Chicago. JAMA Pediatrics. 2024;178(5):473-9.

48. Martinez FD, Wright AL, Taussig LM, Holberg CJ, Halonen M, Morgan WJ. Asthma and wheezing in the first six years of life. The Group Health Medical Associates. N Engl J Med. 1995;332(3):133-8.

49. Bloomberg GR. Recurrent wheezing illness in preschool-aged children: assessment and management in primary care practice. Postgrad Med. 2009;121(5):48-55.
